# The Effect of Platelet Dose on Outcomes after Platelet Rich Plasma Injections for Musculoskeletal Conditions: A Systematic Review and Meta-Analysis

**DOI:** 10.1007/s12178-024-09922-x

**Published:** 2024-09-27

**Authors:** William Berrigan, Frances Tao, Joel Kopcow, Anna L. Park, Isabel Allen, Peggy Tahir, Aakash Reddy, Zachary Bailowitz

**Affiliations:** 1 Department of Orthopaedic Surgery, University of California, 1500 Owens Street, San Francisco, 94158 USA; 2 Department of Family & Community Medicine, University of California, San Francisco, USA; 3 School of Medicine, University of California, San Francisco, USA; 4 Department of Epidemiology and Biostatistics, University of California, San Francisco, USA; 5 University of California, Berkeley, CA USA; 6 Kaiser Permanente Oakland Medical Center, Oakland, CA USA; 7 Kaiser Permanente Bernard J. Tyson School of Medicine, Pasadena, CA USA

**Keywords:** Platelet rich plasma (PRP), Platelet dosing, Platelet dosage, Osteoarthritis (OA), Tendinopathy

## Abstract

**Purpose of Review:**

This study aims to systematically review platelet dosage in platelet rich plasma (PRP) injections for common musculoskeletal conditions.

**Recent Findings:**

Notable heterogeneity exists in the literature regarding platelet dosage. Clinical studies indicate that a higher dosage may lead to improved outcomes concerning pain relief, functional improvement, and chondroprotection in knee osteoarthritis (OA). However, the impact of dosing on other musculoskeletal pathologies remains uncertain. Our investigation identifies a potential dose-response relationship between platelet dose and PRP effectiveness for knee OA treatment, pinpointing an optimal threshold of greater than 10 billion platelets for favorable clinical outcomes. Notably, this effect appears more pronounced for functional outcomes than for pain relief. For other conditions, a lower dosage may suffice, although the existing literature lacks clarity on this matter.

**Summary:**

PRP dosage may significantly influence treatmentoutcomes, particularly in knee OA. Further research is warranted to elucidate optimal dosages for varying conditions.

**Supplementary Information:**

The online version contains supplementary material available at 10.1007/s12178-024-09922-x.

## Introduction

Platelet Rich Plasma (PRP) is an autologous mixture of concentrated platelets and growth factors that is prepared through centrifugation of whole blood. PRP injections have become increasingly popular as a treatment for many musculoskeletal (MSK) conditions. They are most commonly used for osteoarthritis (OA) and tendinopathy, although PRP has also been used in other musculoskeletal conditions such as adhesive capsulitis or as augmentation for fracture healing [1]. Despite its increasing popularity, the efficacy of PRP therapy for many pathologies is still disputed [2].

While PRP is increasingly viewed as a viable nonoperative treatment for OA, consensus on its efficacy and comparison to other treatments remains elusive. Most evidence for the use of PRP in OA comes from studies on knee OA. These data suggest that PRP is equivalent to or better than hyaluronic acid (HA) or corticosteroid injections (CSI); yet many studies are limited by significant heterogeneity in PRP preparation, injection, and post-procedural protocols [2]. Furthermore, it is unclear how generalizable knee OA findings are to outcomes for other joints. Evidence for the use of PRP in tendinopathy remains mixed, with increasing attention to the use of PRP in lateral elbow epicondylopathy, patellar tendinopathy, rotator cuff tendinopathy, and Achilles tendinopathy. Again, conclusions regarding the efficacy of PRP in tendinopathy are limited by heterogeneity in PRP preparation, protocols, and outcome measures.

The process by which PRP is created has substantial inherent variability. Factors such as the amount of blood drawn, centrifugation time, and number of spins can all impact the composition of the final product. Even the definition of PRP itself is debated, as the U.S. Food and Drug Administration’s classification of PRP requires a minimum of 250,000 platelets per microliter, with others suggesting a minimum of 1 million to 1.5 million platelets per microliter [3]. This heterogeneity ultimately leads to differences in platelet, leukocyte, and growth factor composition. The 2017 Minimum Information for Biologics in Orthopedics (MIBO) guidelines define important characteristics for standardizing reporting in studies involving biologics in orthopedic research, including platelet concentration, leukocyte differential, and volume injected [4]. Despite these reporting guidelines, no standardized injection regimens have been established.

Prior studies have called attention to the heterogeneity in PRP literature regarding the composition and preparation of PRP, injection protocols, platelet dosage, and post-injection rehabilitation [2, 5–7]. Platelet dosage is one of many factors that may influence PRP outcomes and has substantial heterogeneity in the literature. Platelet dosage is a measure of the total number of platelets delivered in a PRP treatment and is determined by the product of platelet count (usually in platelets/microliter), volume injected, and total number of injections. A recent review characterizes the importance of platelet dosing in initiating angiogenic pathways necessary for microvascular networks that supply oxygen and nutrients to impaired tissues [8]. However, very few studies have specifically evaluated the impact of platelet dosing on the efficacy of PRP across different pathologies. The purpose of this paper is to systematically review platelet dosage in the literature on PRP injections for musculoskeletal conditions. We hypothesize that higher platelet dosage will be correlated with improved outcomes for PRP injection across pathologies.

## Methods

### Study Selection and Eligibility Criteria

We included studies that investigated the use of platelet rich plasma as the primary treatment for knee OA, hip OA, glenohumeral OA, carpometacarpal joint OA, rotator cuff tendinopathy, patellar tendinopathy, Achilles tendinopathy, gluteal tendinopathy, lateral epicondylopathy, plantar fasciitis, carpal tunnel, cubital tunnel, and ankle osteochondral defects (OCD). We limited our search to randomized clinical trials (RCTs) and prospective or retrospective cohort studies with a minimum of 20 patients that were published in the English language. We excluded meta-analyses, review articles, case reports, case series, conference abstracts, and animal studies. Cohort studies were distinguished from case series according to previous recommendations [9]. PRP associated with a surgical procedure, and PRP used for the treatment of spinal disorders, adhesive capsulitis, and De Quervain’s tenosynovitis were also excluded. Studies that lacked the necessary data to calculate the platelet dosage or had less than 6 months of follow-up were also excluded. Studies were screened by a minimum of 2 independent reviewers, and any discordance on study inclusion was resolved by the principal investigator.

### Search Strategy

We performed comprehensive searches in PubMed, Web of Science, Embase, and the Cochrane Library to obtain articles for our review (Appendix 1). With the assistance of a research librarian, searches were structured to include these main concepts: platelet rich plasma and musculoskeletal pathologies. We developed multiple synonyms for orthopedic terms and conditions including osteoarthritis, tendinopathies, and neuropathies. Searches were constructed to be broad and sensitive. We also developed a third search hedge to include specific types of research studies we were interested in finding. Searches included both keywords and index terms (Mesh and Emtree), depending on the individual database. The searches were conducted on 11/07/2023 and are inclusive of literature from 1/1/2017–11/07/2023. We limited Embase search results to articles and articles in press. Full search strategies for each database are included in the search appendix.

### Data Extraction

Data on the author, publication year, sample size, mean age, mean BMI, comparator, primary and secondary outcome measures, Kellgren-Lawrence classification, MARSPILL classification parameters, preparation kit, injection quantity, mean platelet count, PRP volume injected, total platelet dosage (total platelet dosage x 10^6^= (mean platelet count per microliter x 10^3^) x (volume injected in milliliters) x (total # of injections)), and the resulting p values at 6 months, 1 year, and 2 years were collected using a standardized template [10]. Outcome measure data was included if it was present in 3 or more studies. Primary and secondary outcome data was collected at 6 months, 1 year, and 2 years. The authors were contacted to retrieve any missing data necessary for meta-analysis.

### Data Synthesis and Statistical Analysis

To quantify the effect of PRP compared with other interventions, we calculated mean differences from baseline to 6- and 12-months post-intervention and compared the mean differences and 95% confidence intervals using a random effects meta-analysis to take account of any heterogeneity within and between included studies. Groups of studies were synthesized when there were at least 3 studies in the condition group. Similarly, time points at 6 months and 12 months were synthesized when at least 3 studies in any condition were available for platelet dosages < 5 billion, 5–10 billion, and > 10 billion (1 billion platelets = 1000 × 10^6^ platelets). Heterogeneity was assessed using both Cochran’s Q-statistic and the I-squared statistic. We considered an I-squared greater than 50% indicative of high heterogeneity. Meta-regression was used to identify trends in outcome measures by increasing platelet dosage. Sensitivity analyses were performed to examine potential publication bias, including jackknife (leave out one) analyses and Begg and Egger statistics. All statistical analyses were performed using Stata 18.1 (StataCorp, College Station, TX).

### Risk of Bias Assessment/Quality Assessment

Risk of bias was assessed in all RCTs using the revised Cochrane risk of bias tool, which factors in random sequence generation, allocation concealment, blinding, completeness of outcome data, selective reporting, and other biases [11]. The assessment was completed by two independent reviewers and any discordance was resolved by the principal investigator.

## Results

**Fig. 1:**
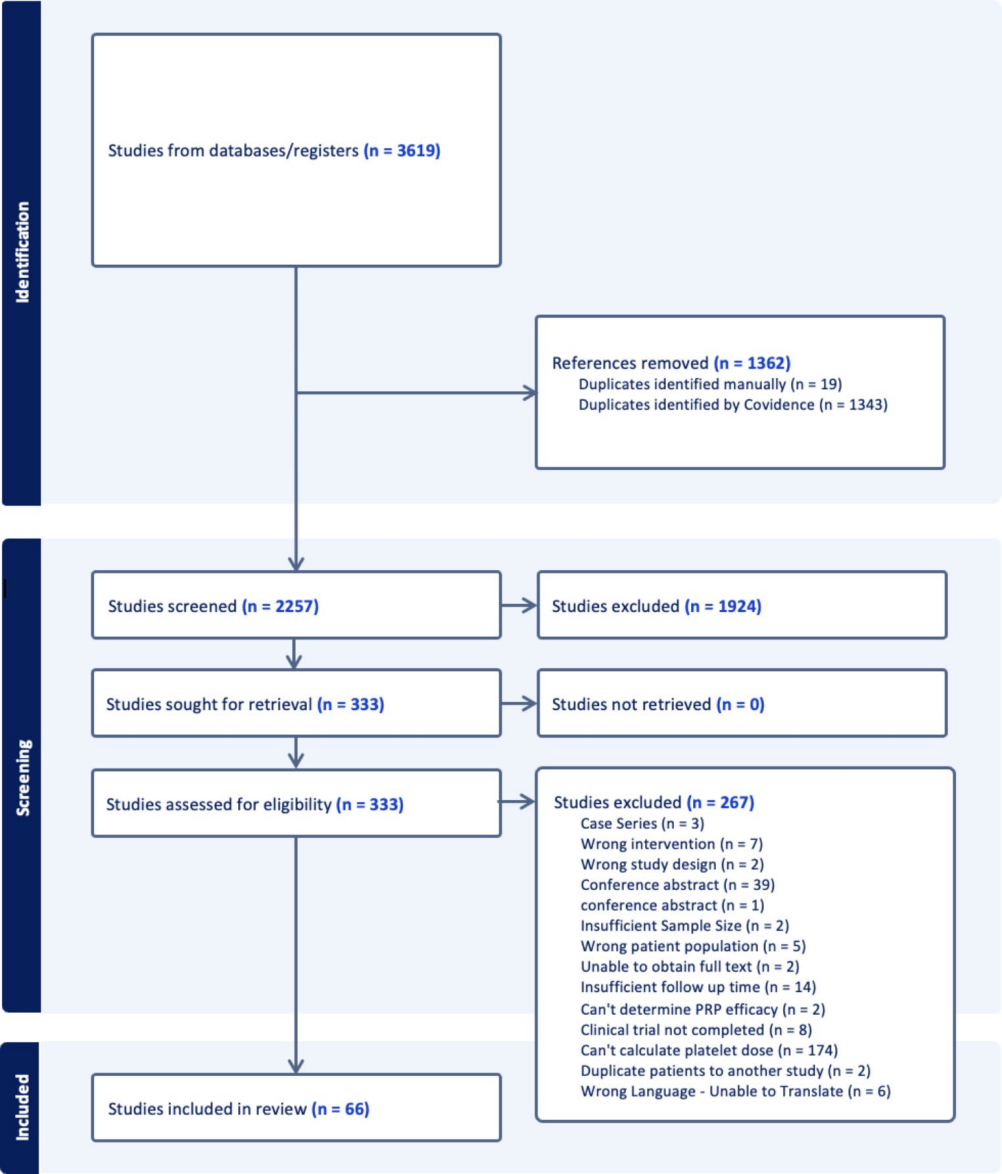
PRISMA (Preferred Reporting Items for Systematic Reviews and Meta-Analyses) flow diagram

After eliminating duplicates, our initial search yielded 2276 studies. Following title and abstract screening for relevance, 336 studies remained. Subsequent full-text screening based on the specified criteria identified 66 studies that were included in our review (Fig. 1). There were 26 level I (40%), 28 level II (42%), and 12 level III (18%) studies. There were 42 studies focused on knee OA, 5 on rotator cuff tendinopathy, 3 on lateral epicondylopathy, 3 on hip OA, 3 on plantar fasciitis, 2 on patellar tendinopathy, 2 on Achilles tendinopathy, 2 on gluteal tendinopathy, 1 on glenohumeral OA, 1 on carpal tunnel syndrome, 1 on carpometacarpal joint OA, 1 on ankle OCD, and 0 for cubital tunnel syndrome. The PRP preparation and treatment protocols for each of these studies are summarized in Table 1. Platelet dosages are reported in billions and multiples of 10^6^ platelets (1 billion platelets = 1000 × 10^6^ platelets).

**Table 1:** PRP Preparation and Treatment protocols

Lead Author (Year)	LOE	Comparator	Method (H/M)	Activation (A+/A-)*	RBC (RBC-*R*/RBC-*P*)*	Spin (Sp1/Sp2)	Platelet Number	Image Guidance*	Leukocyte Concentration (LR/LP*	Light Activation (A+/A-)*	Injection Quantity	Volume injected (mL)	Platelet Average (per microliter) x10^3^	Total Platelet Dosage/injection (x 10^6^)	Total Platelets w/ multiple injections
Glenohumeral Osteoarthritis															
Kirschner et al. (2022) [32]	I	HA	M	A-	RBC-P	Sp2	PL2-3	G+	LP	A-	1	6	680.16	4080.96	4080.96
**Carpometacarpal Osteoarthritis**															
Hasley et al. (2023) [33]	III	N/A	M	A-	RBC-P	Sp2	PL8-10	G+	LP	A-	1	1.1	1781.8	1966.58	1966.58
**Knee Osteoarthritis**															
Akan et al. (2018) [22]	II	Exercise	H	A-	RBC-P	Sp2	PL6-8	G-	LR	A-	3	1	1105	1105	3315
Anz et al. (2022) (66)	II	BMAC	H	A-	RBC-P	Sp2	PL4-6	G+	LP	A-	1	7	1216	8512	8512
Baki et al. (2021) (67)	II	NSAIDs	M	A+	RBC-P	Sp2	PL10+	G-	LP	A-	2	5	2560	12,800	25,600
Bansal et al. (2021) [56]	I	HA	M	A-	RBC-P	Sp2	PL6-8	G-	LP	A-	1	8	1438	10,450	11,504
Baria et al. (2022) (68)	II	MFAT	M	A-	RBC-P	Sp2	PL10+	G+	LR	A-	1	8	2673	13685.76	13685.76
Barman et al. (2022) (69)	I	Intraosseous PRP	H	A-	RBC-P	Sp2	PL4-6	G+	LR	A-	1	8	857.7	6861.6	6861.6
Bec et al. (2021) [23]	III	N/A	M	A+	RBC-P	Sp1	PL1-2	G-	LP	A-	1	6.77	NR	2870	2870
Branch et al. (2023) (70)	II	HA + PRP	M	A-	RBC-P	Sp1	PL1-2	G-	LP	A-	3	4.5	406.32	1828.44	5485.32
Buendia-Lopez et al. (2018) (71)	II	NSAIDs, HA	H	A+	RBC-P	Sp2	PL2-3	G-	LP	A-	1	5	1095	5475	5475
Chu et al. (2022) (72)	I	NS	H	A-	RBC-P	Sp2	PL4-6	G-	LP	A-	3	5	832.1	4160.5	12481.5
Sdeek et al. (2021) (73)	I	HA	H	A-	RBC-P	Sp2	PL8-10	G-	LP	A-	3	2.5	2664	6,660	19,980
Zaffagnini et al. (2022) [16]	II	MFAT	H	A+	RBC-P	Sp2	PL4-6	G-	LR	A-	1	5	1000	5000	5000
Di Martino et al. (2022) (74)	I	LP- vs. LR-PRP	H	A+	RBC-P	Sp2	PL4-6	G-	LP	A-	3	5	1146.8	5734	17,202
									LR		3	5	1074.9	5374.5	16123.5
Dorio et al. (2021) (75)	I	NS, Plasma	H	A-	RBC-P	Sp2	PL4-6	G+	LP	A-	2	3.2	1000	3200	6400
Elik et al. (2020) (76)	II	NS	M	A+	RBC-P	Sp2	PL4-6	G-	LR	A-	3	4	1000	4000	12,000
Ghai et al. (2019) (77)	I	NS	H	A+	RBC-P	Sp2	PL1-2	G-	LP	A-	1	8	310.14	2385	2481.12
Gormelli et al. (2017) (78)	I	NS, HA, Multidose PRP	H	A+	RBC-P	Sp2	PL4-6	G-	LR	A-	3	5	1118	5590	16,770
											1	5	1152	5760	5760
Govila et al. (2023) [24]	II	N/A	H	A-	RBC-P	Sp2	PL4-6	G-	LP	A-	1	4.5	1019	4585.5	4585.5
Guillibert et al. (2019) [25]	II	N/A	M	A-	RBC-P	Sp1	PL1-2	G+	LP	A-	1	8.8	288	2534.4	2534.4
Joshi Jubert et al. (2017) (79)	II	CSI	H	A-	RBC-P	Sp2	PL4-6	G-	LP	A-	1	4	990	3960	3960
Kaszynski et al. (2022) (80)	I	Adipose Tissue	H	A-	RBC-P	Sp1	PL8-10	G-	LP	A-	3	3	1720	5160	15,480
Li et al. (2021) [17]	III	HA	H	A-	RBC-P	Sp2	PL6-8	G-	LR	A-	3	3.5	819.136	2866.976	8600.928
Li et al. (2023) (81)	II	HA	H	A-	RBC-P	Sp2	PL4-6	G-	LR	A-	3	4	826.37	3305.48	9916.44
Louis et al. (2018) (82)	II	HA	H	A+	RBC-P	Sp2	PL2-3	G+	LP	A-	1	3	800	2400	2400
Montanez-Heredia et al. (2016) [12]	I	HA	H	A+	RBC-P	Sp2	PL6-8	G-	LP	A-	3	5	952	4760	14,280
Nunes-Tamashiro et al. (2022) (83)	I	CSI	H	A-	RBC-P	Sp1	PL4-6	G-	NR	A-	1	6	1119.588	6717.528	6717.528
Palco et al. (2021) [28]	III	HA + PRP	M	A-	RBC-P	Sp1	PL1-2	G-	LR	A-	1	5	290	1450	1450
Park et al. (2021) (84)	I	HA	M	A+	RBC-P	Sp1	PL4-6	G-	LR	A-	1	3	976	2928	2928
Saita et al. (2021) [18]	III	N/A	M	A-	RBC-P	Sp1	Pl2-3	G-	LP	A-	3	4.5	475.4	2139.3	6417.9
Sanchez et al. (2022) [19]	II	N/A	H	A+	RBC-P	Sp1	PL1-2	G-	LP	A-	3	8	309	2472	7416
Sandhu et al. (2022) [13]	II	N/A	M	A+	RBC-P	Sp2	PL4-6	G-	LP	A-	3	8	1000	8000	24,000
Saqlain et al. (2021) [14]	II	Multidose PRP	H	A-	RBC-P	Sp2	PL4-6	G-	LP	A-	1	5	1100	5500	5500
											2	5	1100	5500	11,000
Silvestre et al. (2023) [15]	III	N/A	M	A-	RBC-P	Sp1	PL2-3	G+	LP	A-	2	10	573	5730	11,460
Simental-Mendia et al. (2019) [21]	II	Multidose PRP	H	A+	RBC-P	Sp2	PL2-3	G-	LP	A-	1	5	499.3	2496.5	2496.5
											3	5	446.3	2231.5	6694.5
Singh et al. (2022) (85)	II	Arthroscopy	H	A-	RBC-P	Sp1	PL4-6	G-	LP	A-	1	4.5	1019	4585.5	4585.5
Su et al. (2018) (86)	II	HA, Intraosseous PRP	H	A+	RBC-P	Sp2	PL4-6	G-	LR	A-	2	6	789.68	4738.08	9476.16
Subramanyam et al. (2021) [26]	I	Multidose PRP	M	A-	RBC-P	Sp1	PL2-3	G-	LP	A-	1	4	392	1568	1568
											2	4	386	1544	3088
											3	4	386	1544	4632
Sun et al. (2021) (87)	I	HA + PRP	M	A-	RBC-P	Sp1	PL2-3	G-	LP	A-	1	3	463.83	1391.49	1391.49
Taniguchi et al. (2018) [20]	II	N/A	H	A-	RBC-P	Sp1	PL1-2	G-	LP	A-	3	6	394	2364	7092
Tucker et al. (2021) [27]	II	NS	M	A-	RBC-P	Sp1	PL2-3	G+	LP	A-	1	5	703.73	3518.65	3518.65
Wang et al. (2022) [29]	I	HA	H	A+	RBC-P	Sp2	PL6-8	G-	LR	A-	1	4	857.4	3429.6	3429.6
Xu et al. (2021) (88)	I	HA, HA + PRP	H	A-	RBC-P	Sp2	PL4-6	G+	LP	A-	3	4	950	3800	11,400
**Hip Osteoarthritis**															
Nouri et al. (2022) [30]	I	HA, HA + PRP	M	A-	RBC-P	Sp2	PL4-6	G+	LP	A-	2	5	1402.03	7010.15	14020.3
Palco et al. (2021) [28]	III	HA + PRP	M	A-	RBC-P	Sp1	PL1-2	G+	LR	A-	1	5	370	1850	1850
Villanova-Lopez et al. (2020) [31]	III	HA	H	A+	RBC-P	Sp1	PL2-3	G+	LP	A-	1	6	586.21	3517.26	3517.26
**Epicondylopathy**															
Alessio-Mazzola et al. (2018) [36]	III	ECSW	H	A-	RBC-P	Sp3	PL2-3	G+	LP	A-	4	3	600	1800	7200
Gupta et al. (2020) [35]	II	CSI	H	A-	RBC-P	Sp2	PL4-6	G-	LP	A-	1	3	823	2469	2469
Lim et al. (2018) [34]	II	PT	M	A+	RBC-P	Sp1	PL6-8	G+	LR	A-	1	5	1890	9450	9450
**Rotator Cuff Tendinopathy**															
Cai et al. (2019) [39]	I	NS, HA, HA + PRP	H	A-	RBC-P	Sp2	PL4-6	G+	LP	A-	4	4	1000	4000	16,000
Hewavithana et al. (2023) [41]	I	CSI	M	A-	RBC-P	Sp1	PL2-3	G+	LP	A-	1	6	654	3924	3924
Jo et al. (2020) [40]	I	CSI	M	A+	RBC-P	NR	PL4-6	G+	LP	A-	1	4	989	3956	3956
Nejati et al. (2017) [38]	II	Exercise	M	A-	RBC-P	Sp2	PL2-3	G+	LR	A-	2	4	900	3600	7200
Rossi et al. (2021) [37]	II	N/A	H	A-	RBC-P	Sp2	PL4-6	G-	LR	A-	1	5	1004	5020	5020
**Gluteal Tendinopathy**															
Fitzpatrick et al. (2019) [42]	I	CSI, CSI + PRP	M	A-	RBC-P	Sp1	PL4-6	G+	LR	A-	1	6.5	964	6266	6266
Thompson et al. (2019) [43]	I	NS	M	A-	RBC-P	Sp1	PL4-6	G-	LP	A-	1	5	1232.3	6161.5	6161.5
**Patellar Tendinopathy**															
Rodas et al. (2021) [45]	I	BM-MSCs	H	A+	RBC-P	Sp1	PL2-3	G+	LP	A-	2	6	563	3378	6756
Scott et al. (2019) [44]	I	NS, LP- vs. LR-PRP	M	A-	RBC-P	Sp1	PL2-3	G+	LR	A-	1	3.5	874	3059	3059
									LP		1	3.5	681	2383.5	2383.5
**Achilles Tendinopathy**															
Erroi et al. (2017) [46]	III	ECSW	M	A-	RBC-P	Sp1	PL4-6	G+	LP	A-	2	2	995	1990	3980
Usuelli et al. (2018) [47]	II	SVF	M	A-	RBC-P	Sp1	PL4-6	G+	LP	A-	1	4	813	3252	3252
**Plantar Fasciitis**															
Alessio-Mazzola et al. (2023) [51]	III	ECSW	H	A-	RBC-P	Sp3	PL2-3	G+	LP	A-	3	3	600	1800	5400
Soraganvi et al. (2019) [49]	I	CSI	H	A-	RBC-P	Sp2	PL4-6	G-	LP	A-	1	3	1000	3000	3000
Srivastava et al. (2022) [50]	II	CSI	H	A-	RBC-P	Sp1	PL4-6	G-	LP	A-	1	3	1000	3000	3000
**Carpal Tunnel Syndrome**															
Uzun et al. (2017) [48]	II	CSI	H	A-	RBC-P	Sp1	PL6-8	G-	LP	A-	1	2	1532	3064	3064
**Ankle OCD**															
Akpancar et al. (2019) [52]	III	Prolotherapy	M	A-	RBC-P	Sp1	PL4-6	G+	LP	A-	3	4	1007	4028	12,084

HA: Hyaluronic Acid, BMAC: Bone Marrow Aspirate Concentrate, NSAIDS: Non-steroidal Anti-inflammatory Drug, MFAT: Micro-fragmented Adipose Tissue, PRP: Platelet-rich plasma, NS: Normal Saline, CSI: Corticosteroid Injection, ECSW: Extracorporeal Shock Wave, PT: Physical Therapy, BM-MSC: Bone Marrow-derived Mesenchymal Stem Cells, SVF: Stromal Vascular Fraction, N/A: Not applicable, LOE: Level of evidence, LR: leukocyte-rich PRP, LP: leukocyte-poor PRP, PRP: Platelet-rich plasma, RBC-P: Poor red blood cells, RBC-R: Rich red blood cells. *In instances where information on activation, RBC-Poor vs. RBC-Rich, image guidance, Leukocyte-Poor vs. Leukocyte-Rich, and light activation was not provided, the assessment was noted as poor or negative

### Risk of Bias Assessment

**Fig. 2:**
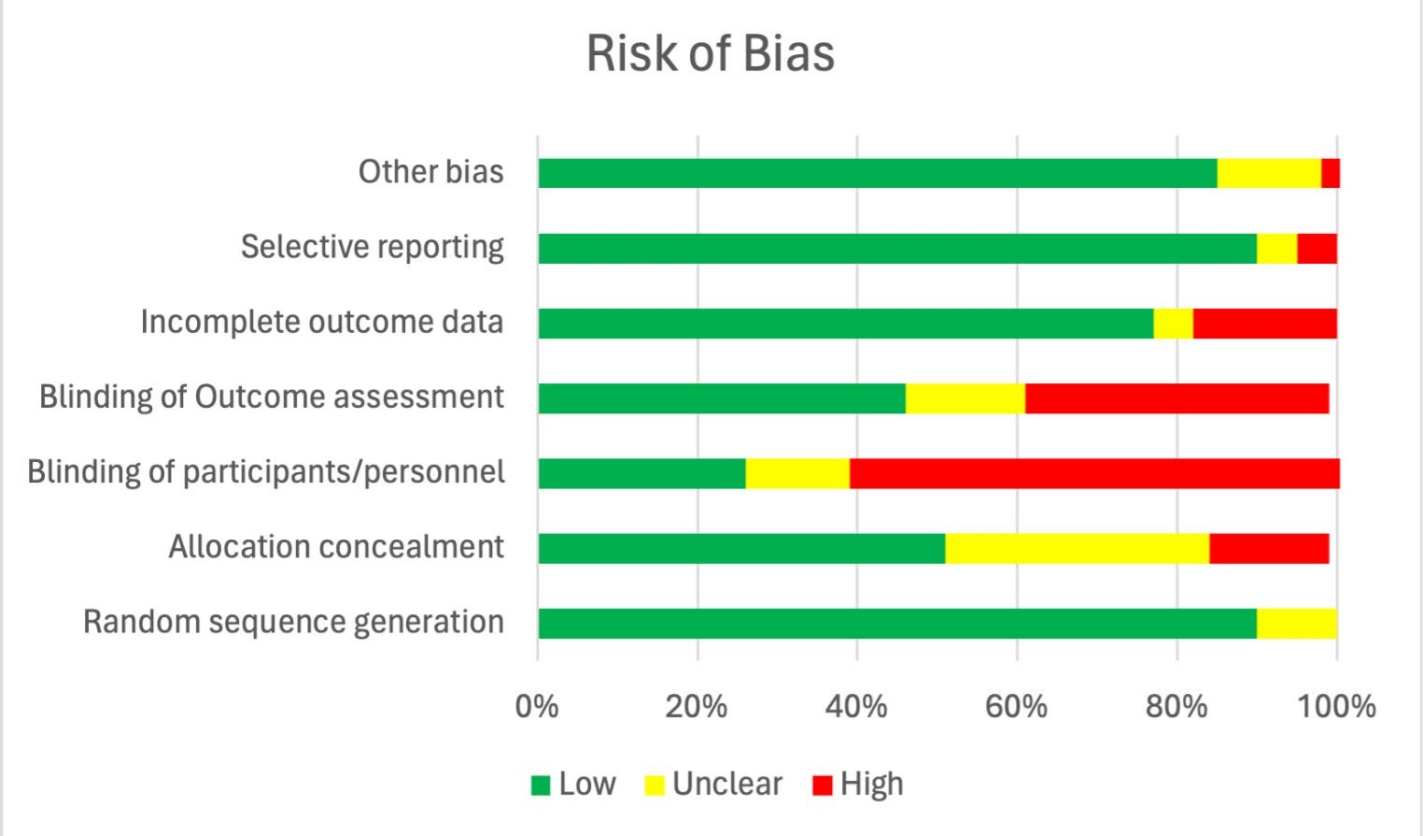
Risk of Bias graph. risk of bias is presented as a percentage across all included RCT studies

Overall, the risk of bias was low among 40 RCTs (Fig. 2). The risk of bias was highest for blinding participants, personnel, and outcome assessors. Among the remaining studies, the main concerns for bias were due to allocation concealment and incomplete outcome data.

### Osteoarthritis (OA)

The platelet dosage and efficacy of PRP for the treatment of osteoarthritis at 6 months, 1 year, and 2 years post-injection are summarized in Table 2.

**Table 2:** Summary of platelet dosage and efficacy of prp for treatment of osteoarthritis at 6 months, 1 year, and 2 years post-injection

Author (Yr)	Comparator	Total Platelet Dose	Efficacy (6 m)	Relative to Comparator (6 m)	Efficacy (1y)	Relative to Comparator (1y)	Efficacy (2y)	Relative to Comparator (2y)
Hip Osteoarthritis								
Palco et al. (2021) [28]	HA + PRP	1850 × 10^6^	N/A	N/A	-	N/A	N/A	N/A
Villanova-Lopez et al. (2020) [31]	HA	3517 × 10^6^	N/A	-	+	-	N/A	N/A
Nouri et al. (2022) [30]	HA, HA + PRP	14,020 × 10^6^	+	+	N/A	N/A	N/A	N/A
**Glenohumeral Osteoarthritis**								
Kirschner et al. (2022) [32]	HA	4081 × 10^6^	+	-	+	-	N/A	N/A
**Carpometacarpal Osteoarthritis**								
Hasley et al. (2023) [33]	N/A	1967 × 10^6^	N/A	N/A	+	N/A	N/A	N/A

HA: Hyaluronic acid, PRP: Platelet-rich plasma, N/A: Not applicable

### Knee OA

**Fig. 3:**
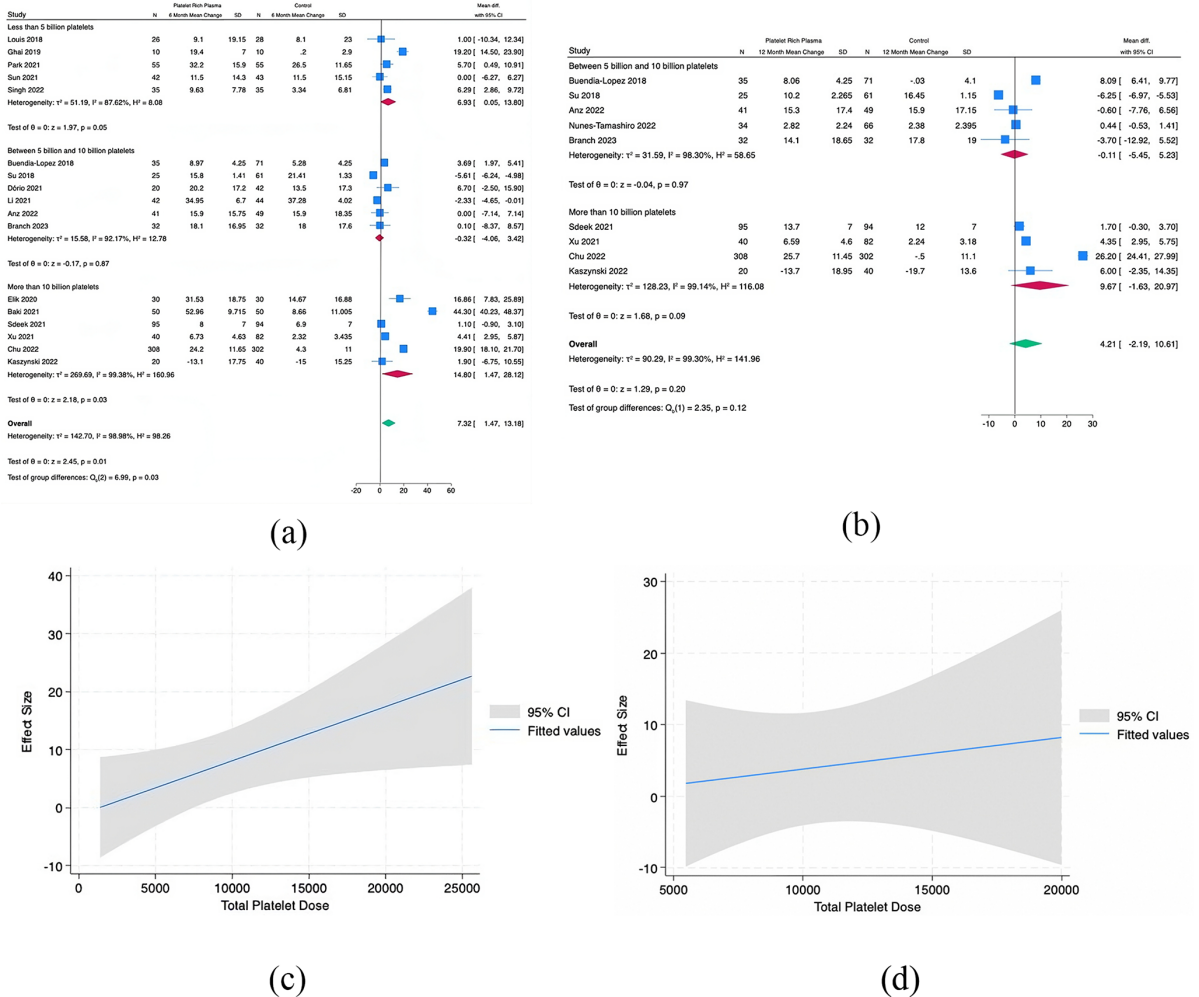
(**A**) Pooled Analysis of Baseline to 6 months in WOMAC for PRP vs. Control by Total Platelet Subgroups, (**B**) Pooled Analysis of Baseline to 12 months in WOMAC for PRP vs. Control by Total Platelet Subgroups, (**C**) Meta Regression Analysis for WOMAC at 6 months, and (**D**) Meta Regression Analysis for WOMAC at 12 months. (Platelet Dose x10^6^)

**Fig. 4:**
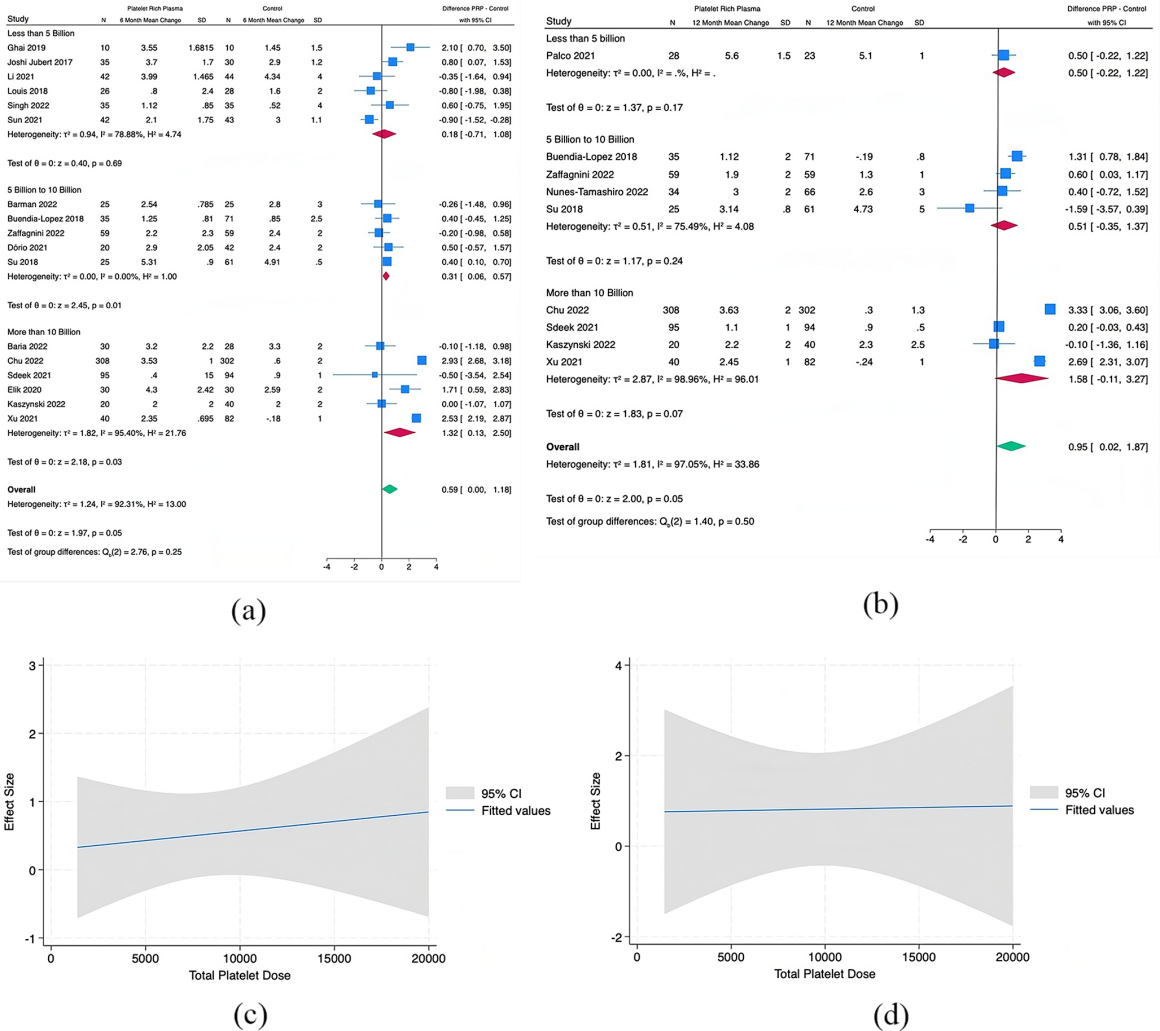
(**A**) Pooled Analysis of Baseline to 6 months in VAS for PRP vs. Control by Total Platelet Subgroups, (**B**) Pooled Analysis of Baseline to 12 months in VAS for PRP vs. Control by Total Platelet Subgroups, (**C**) Meta Regression Analysis for VAS at 6 months, and (**D**) Meta Regression Analysis for VAS at 12 months. (Platelet Dose x10^6^)

**Fig. 5:**
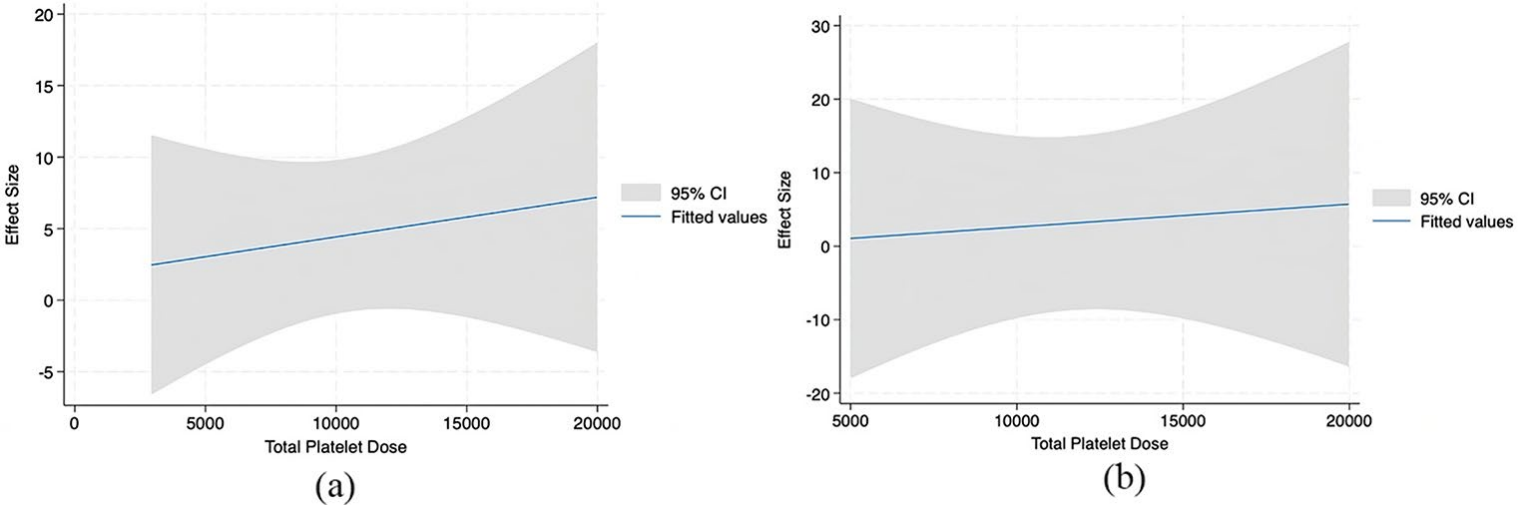
Meta Regression Analysis for IKDC at (A) 6 months and (B) 12 months

**Fig. 6:**
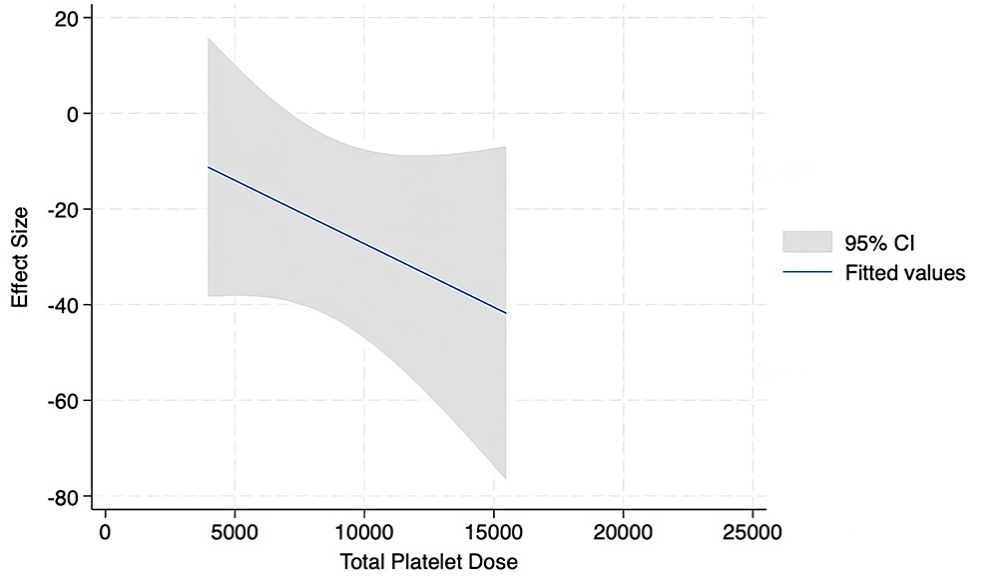
Meta Regression Analysis for KOOS Sport at 6 months

24 studies were included in the meta and regression analysis. 17 reported WOMAC (Figs. 3), 17 reported VAS (Figs. 4), 10 reported IKDC (Fig. 5), and 6 reported KOOS (Fig. 6).

### Western Ontario and McMaster Universities Osteoarthritis Index (WOMAC)

Pooled analysis from 5 studies with a dose of < 5 billion platelets and mean latest follow-up of at least 6 months demonstrated that the PRP group had a significant difference in subjective WOMAC scores than the comparator groups (Mean Difference (MD), 6.93 [95% CI, 0.05-13.8]; *p* = 0.05). Analysis from 6 studies with 5–10 billion platelets demonstrated no difference between comparators (MD, -0.32 [95% CI, -4.06,3.42]; *p* = 0.87). Six Studies with > 10 billion platelets demonstrated a difference in favor of PRP (MD, 14.8 [95% CI, 1.47–28.12]; *p* = 0.03). The *I*^2^ statistic for WOMAC scores was 87%, 92%, and 99% for < 5 billion, 5–10 billion, and > 10 billion platelets respectively.

No studies with 12-month data used < 5 billion platelets. Pooled analysis from 5 studies with 5–10 billion platelets and mean latest follow-up of at least 12 months demonstrated no difference between comparators (MD, -0.11 [95% CI, -5.45-5.23]; *p* = 0.97). Four studies with > 10 billion platelets demonstrated a near difference in favor of PRP (MD, 9.67 [95% CI, -1.63-20.97]; *p* = 0.09). The *I*^2^ statistic for WOMAC scores was 98% and 99% for 5–10 billion, and > 10 billion platelets respectively.

Meta-regression analysis at 6 months showed a significant trend and demonstrated that larger decreases in WOMAC scores were seen with higher doses of platelets. At 12 months, a consistent trend of greater effect size with increasing platelet dose was seen but the slope was lower than at the 6-month time point (Appendix 2).

No study bias was seen in the Begg and Egger statistics, and a jackknife analysis leaving out each study and recalculating the results showed no change in the overall summary effect sizes.

### Visual Analog Scale (VAS)

Pooled analysis from 6 studies with a dose of < 5 billion platelets and mean latest follow-up of at least 6 months demonstrated that the PRP group had no difference in subjective VAS scores than the comparator groups (MD, 0.18 [95% CI, -0.71-1.08]; *p* = 0.69). Analysis from 5 studies with 5–10 billion demonstrated a difference that favored PRP to the comparators (MD, 0.31 [95% CI, 0.06–0.57]; *p* = 0.01). Six studies with > 10 billion platelets demonstrated a difference in favor of PRP (MD, 1.32 [95% CI, 0.13–2.50]; *p* = 0.03). The *I*^2^ statistic for VAS pain scores was 78%, 0%, and 95% for < 5 billion, 5–10 billion, and > 10 billion respectively.

One study with a dose of < 5 billion platelets and latest follow-up of at least 12 months demonstrated that the PRP group had no difference in subjective VAS scores than the comparator group (MD, 0.5 [95% CI, -0.22-1.22]; *p* = 0.17). Pooled analysis from 4 studies with 5–10 billion demonstrated no difference between PRP and the comparators (MD, 0.51 [95% CI, -0.35-1.37]; *p* = 0.24). Four studies with > 10 billion platelets demonstrated a near difference in favor of PRP (MD, 1.58 [95% CI, -0.11-3.27]; *p* = 0.07). The *I*^2^ statistic for VAS pain scores was 75%, and 97% for 5–10 billion, and > 10 billion platelets respectively.

Meta-regression analysis demonstrated a slight trend at 6 months with increasing effect (as measured by VAS) with an increasing number of platelets. No trend was seen at the 12-month evaluation (Appendix 2).

### IKDC

There were insufficient studies to run meta-analysis by dosages for the IKDC. Meta-regression analysis at 6 and 12 months for IKDC revealed a significant trend of increased effectiveness with an increasing number of total platelets (Appendix 2).

### KOOS

There were insufficient studies to run meta-analysis by dosages for the KOOS. Meta-regression analysis at 6 months for KOOS Sport revealed a trend in significantly decreasing symptoms with an increasing number of total platelets (Appendix 2).

Other Knee OA Studies.

18 knee OA clinical studies were not included in the meta and regression analysis. Out of 4 studies that administered PRP with a dose > 10 billion platelets, 3 reported positive outcomes for the PRP group at 6 months post-injection [12–14], and 2 reported positive outcomes at 1 year post-injection [13, 15]. 7 studies had a total PRP dose between 5 and 10 billion platelets. Six of these studies reported positive outcomes for the PRP group at 6 months post-injection [14, 16–20]. Li et al. reported outcomes for the PRP group that were significantly superior to the comparator group at 6 months and 1 year follow-up. Additionally, positive outcomes for the PRP group were reported at 1-year post-injection for 4 studies [16, 18, 19, 21], and at 2 years post-injection for 1 study [16]. The remaining 9 studies administered PRP with a total dose of < 5 billion platelets. 6 studies reported positive outcomes for the PRP group at 6 months post-injection [22–27], and 3 studies reported positive outcomes at 1 year post-injection [21, 26, 28]. Two studies reported outcomes for the PRP group that were significantly superior to the comparator group at 6 months follow-up [22, 29].

### Hip OA

Three studies examined the effect of PRP on hip osteoarthritis. A high-quality RCT compared the effectiveness of three treatment arms: HA, leukocyte-poor PRP (LP-PRP), and the combination of HA with LP-PRP [30]. Every group received 2 injections separated by 2 weeks. The PRP group had a total platelet dosage of 14,020 × 10^6^. All three groups showed significant improvement in total WOMAC scores at 6 months post-injection (*p* < 0.001). The authors found a significant difference in total WOMAC scores favoring PRP to HA (*p* = 0.022), as well as favoring the combination of PRP with HA to HA alone (*p* = 0.007). There was, however, no statistical difference when comparing the combination of PRP with HA to PRP alone.

A retrospective study compared leukocyte-rich PRP (LR-PRP) to a combination injection of both LR-PRP and HA [28]. The total platelet dosage in the PRP injections was 1850 × 10^6^. At one year’s follow-up, there was no significant improvement in Harris hip score (HHS) in either group when compared to baseline scores (*p* > 0.05).

The last study was a low-quality double-blinded RCT [31]. The authors found that the LP-PRP group (total platelet dosage of 3517 × 10^6^) had significant improvement in the WOMAC scores at 1 year (*p* < 0.01), however, there was no difference in scores when compared to the HA group at 6 months or 1-year post-injection (*p* = 0.95 and *p* = 0.27 respectively).

### Glenohumeral OA

One double-blinded RCT of 70 patients with a low risk of bias compared a single injection of PRP with a dose of 4081 × 10^6^ platelets to HA [32]. Improvement was noted in both groups at 2, 3, 6, and 12 months after injection but there was no statistically significant difference between the two. The improvements were observed regardless of osteoarthritis severity.

### Carpometacarpal OA

One retrospective cohort study of 19 patients who received a single injection of PRP with a total platelet dosage of 1967 × 10^6^ reported moderate or excellent symptomatic improvement in 68.8% of patients [33]. The mean reported duration of benefit was 15.6 months. The concentration factor for the platelets in the study was 8.8 (+/-4.9).

### Tendinopathy

The platelet dosage and efficacy of PRP for treatment of tendinopathy at 6 months, 1 year, and 2 years post-injection are summarized in Table 3.

**Table 3:** Summary of platelet dosage and efficacy of prp for treatment of tendinopathy at 6 months, 1 year, and 2 years post-injection

Author (Yr)	Comparator	Total platelet dose	Efficacy (6 m)	Relative to comparator (6 m)	Efficacy (1y)	Relative to comparator (1y)	Efficacy (2y)	Relative to comparator (2y)
Epicondylopathy								
Gupta et al. (2020) [35]	CSI	2469 × 10^6^	N/A	N/A	+	+	N/A	N/A
Alessio-Mazzola et al. (2018) [36]	ECSW	7200 × 10^6^	N/A	N/A	N/A	N/A	+	-
Lim et al. (2018) [34]	PT	9450 × 10^6^	+	+	N/A	N/A	N/A	N/A
**Rotator Cuff Tendinopathy**								
Hewavithana et al. (2023) [41]	CSI	3924 × 10^6^	N/A	-	N/A	-	N/A	N/A
Jo et al. (2020) [40]	CSI	3956 × 10^6^	+	-	N/A	N/A	N/A	N/A
Rossi et al. (2021) [37]	N/A	5020 × 10^6^	N/A	N/A	+	N/A	N/A	N/A
Nejati et al. (2017) [38]	Exercise	7200 × 10^6^	+	-	N/A	N/A	N/A	N/A
Cai et al. (2019) [39]	NS, HA, HA + PRP	16,000 × 10^6^	+	+	+	+	N/A	N/A
**Gluteal Tendinopathy**								
Thompson et al. (2019) [43]	NS	6161 × 10^6^	+	-	+	-	N/A	N/A
Fitzpatrick et al. (2019) [42]	CSI, CSI + PRP	6266 × 106	+	+	+	N/A	+	N/A
**Patellar Tendinopathy**								
Scott et al. (2019) [44]	NS	2384 × 10^6^ (LR), 3059 × 10^6^ (LP)	+	-	+	-	N/A	N/A
Rodas et al. (2021) [45]	BM-MSCs	6756 × 10^6^	+	-	N/A	N/A	N/A	N/A
**Achilles Tendinopathy**								
Usuelli et al. (2018) [47]	SVF	3252 × 10^6^	+	-	N/A	N/A	N/A	N/A
Erroi et al. (2017) [46]	ECSW	3980 × 10^6^	+	-	N/A	N/A	N/A	N/A

CSI: Corticosteroid injection, ECSW: Extracorporeal shock wave, PT: Physical therapy, N/A: Not applicable, NS: Normal saline, HA: Hyaluronic acid, LR: Leukocyte-rich PRP, LP: Leukocyte-poor PRP, BM-MSCs-Bone marrow mesenchymal stem cells, SVF-Stromal vascular fraction

### Lateral Epicondylopathy

Three articles studied the use of PRP in lateral epicondylopathy and all of them utilized the VAS as the primary outcome measure. In a single-center, prospective RCT, physical therapy was compared to a single injection of LR-PRP [34]. The PRP had a total platelet dosage of 9450 × 10^6^. The authors found improvement in VAS and functional scores as well as MRI severity grade at 6 months follow-up for the PRP group. Additionally, there was a statistically significant difference between the two treatment groups favoring PRP (*p* < 0.05). Another RCT compared landmark-guided CSI to LP-PRP (total platelet dosage 2469 × 10^6^) for the treatment of chronic refractory lateral epicondylopathy [35]. At one year post-injection, the PRP group significantly outperformed the CSI group in terms of reduction in VAS scores (*p* = 0.024).

A retrospective study comparing LP-PRP to extracorporeal shock wave therapy (ECSW) found significant improvement in VAS scores at 2 years post-injection for the PRP group (*p* < 0.001) [36]. However, there was no difference in results as compared to the ECSW group (*p* > 0.05). The PRP group in this study received four injections for a total platelet dosage of 7200 × 10^6^.

### Rotator Cuff Tendinopathy

Five studies investigated the role of PRP in the treatment of rotator cuff tendinopathy. One prospective cohort study investigated the effects of subacromial PRP injections administered to 50 adults with chronic rotator cuff tendinopathy that was refractory to conservative management [37]. The LR-PRP had a total platelet dosage of 5020 × 10^6^ per injection and was injected via anatomical landmarks. A second PRP injection was administered for 6 (12%) patients at the 3-month follow-up if there was no significant improvement in pain and functional scores, and new injuries were ruled out by MRI. At 12 months follow-up, there was a significant improvement in VAS, Constant, and the American Shoulder and Elbow Surgeons (ASES) scores (*p* < 0.001 for all).

One RCT compared exercise therapy to PRP for the treatment of subacromial impingement. Patients in the PRP group received two injections of LR-PRP (total platelet dosage of 7200 × 10^6^) that were spaced one month apart [38]. A portion of each PRP injection was injected intra-tendinous under ultrasound guidance, while the remainder of the injectate was directed at the subacromial space using landmark guidance. The PRP group achieved a significant reduction in VAS pain scores at 6 months post-injection (*p* < 0.01), however, there was no difference between treatment arms.

In a double-blinded RCT, patients were randomized to receive 4 weekly injections of either normal saline, sodium hyaluronate, LP-PRP, or the combination of sodium hyaluronate with PRP [39]. The total platelet dosage after 4 injections was 16,000 × 10^6^. All injections were completed under ultrasound guidance into the subacromial space. The sodium hyaluronate, PRP, and the combination sodium hyaluronate with PRP groups all had significant improvement in ASES, Constant, and VAS scores at 3, 6, and 12 months when compared to baseline (*p* < 0.05 for all). The PRP and the combination of sodium hyaluronate with PRP groups both showed significantly greater improvements in these pain and functional scores over the same follow-up time intervals as compared to normal saline and sodium hyaluronate groups (*p* < 0.01 for all).

Two RCTs compared LP-PRP injections to CSIs into the subacromial space. In one double-blinded study, the PRP had a total platelet dosage of 3956 × 10^6^. At 6 months follow-up, the Constant score improved significantly above baseline in the PRP group, however, there was no difference between the two treatment arms [40]. In the other RCT, the PRP had a total platelet dosage of 3924 × 10^6^ [41]. The reduction in pain severity as measured by the Neer Pain score was not statistically different between the two treatment arms at 6 months or 1-year post injection (*p* = 0.155 and *p* = 0.081 respectively). There was, however, a significant difference in improvement of shoulder abduction at 12 months follow up favoring the PRP group (*p* = 0.012).

### Gluteal Tendinopathy

Two RCTs assessed the efficacy of PRP in gluteal tendinopathy. One RCT of 80 patients with some concern for risk of bias compared a CSI to a single injection of PRP with a platelet dose of 6266 × 10^6^ [42]. The PRP group improved significantly in the Modified Harris Hip Score (MHHS) from baseline (53.77; SD, 12.08) to 12 (74.05; SD, 13.92), 24 (77.6; SD, 11.88), 52 (78.18; SD, 14.53) and 104 weeks (82.59; SD, 9.71) versus the CSI at 24 weeks (65.82; SD, 15.28). In this study, there was a crossover group for which 27 patients were deemed to have failed CSI with an exit score of 59.22 (SD,11.54). The crossover group improved with the LR-PRP: from 59.22 (SD, 11.22) at baseline to 75.55 (SD, 16.05) at 12 weeks, 77.69 (SD, 15.30) at 24 weeks, and 77.53 (SD, 14.54) at 104 weeks. Another RCT of 48 patients with a high risk of selective reporting bias compared a single injection of PRP with a dosage of 6161 × 10^6^ to normal saline [43]. There was a reduction in worst, average, and least pain over time, but no difference between the two groups at 3,6, or 12 months. The injections in this study were done without the use of image guidance.

#### Patellar Tendinopathy

Two RCTs examined the outcomes in patients with patellar tendinopathy after receiving PRP. In both studies, the primary outcome was the Victorian Institute of Sport Assessment for Pain (VISA-P). One RCT studied single-dose LR-PRP versus LP-PRP versus saline injections [44]. Platelet dosages used in this study were 3059 × 10^6^ and 2384 × 10^6^ in the LP-PRP and LR-PRP groups respectively. Though VISA-P scores improved at 6 months and 1-year post-injection in both PRP groups, the authors found no difference in mean change in VISA-P scores among all treatment groups (*p* > 0.05 for all outcomes). In the other RCT, which was double-blinded, outcomes were compared between those who received two injections of LP-PRP (total platelet dosage 6756 × 10^6^) versus bone marrow mesenchymal stem cells [45]. The PRP group experienced significant improvement in VISA-P scores from baseline (47.00, SD 9.83) to 6 months post-treatment (72.90, SD 17.34; *p* = 0.0009), however, there was no statistical difference in scores between the two treatment arms (*p* = 0.6776).

### Achilles Tendinopathy

Two studies investigated the efficacy of LP-PRP in Achilles tendinopathy using the Victorian Institute of Sport Assessment for Achilles (VISA-A) as the primary outcome measure. A retrospective study investigated the difference in patient outcomes in those with insertional Achilles tendinopathy who received either two injections of LP-PRP (total platelet dosage 3980 × 10^6^) or three sessions of ECSW [46]. The PRP group experienced significant improvement in VISA-A scores at 6 months (82.0, SD 18.1) as compared to baseline (52.8, SD 14.2; *p* < 0.001), but there was no statistical difference in improvement when compared to the ECSW group (*p* = 0.368). Similarly, in an RCT comparing LP-PRP to adipose-derived stromal vascular fraction (SVF) injection, there was no statistical difference between groups at 6 months post-intervention (*p* > 0.05), though VISA-A scores improved significantly from baseline (*p* < 0.001) [47]. The platelet dosage used in this study was 3252 × 10^6^.

#### Plantar Fasciitis

The efficacy of PRP in plantar fasciitis was investigated in three studies. All three studies utilized the VAS as the primary outcome measure. Two of the three studies were RCTs that compared single landmark-guided PRP injection to CSI, and both RCTs injected PRP with a total platelet dosage of 3000 × 10^6^ [48, 49]. In one of the RCTs, not only did the PRP group report improvement in VAS scores at 6 weeks, 3 months, and 6 months post-injection, but there was also a significant difference in VAS scores between the two treatment arms at all three-time intervals (*p* < 0.007, *p* < 0.001, *p* < 0.001 at 6 weeks, 3 months, and 6 months respectively) favoring the PRP group [48]. The PRP group also had a significant reduction in plantar fascia thickness as assessed by ultrasound 6 months post-injection when compared to the corticosteroid group (*p* < 0.0003). The authors of the second RCT found similar results though their study was of lower quality evidence. Both PRP and CSI groups found improvement in VAS scores at 6 weeks, 3 months, and 6 months post-injection [49]. When compared between the two groups, there was a significant reduction in VAS scores that favored the PRP group at all time intervals. Moreover, the PRP group experienced a larger sonographic reduction in plantar fascia thickness at 6 months post-injection as compared to the CSI group (*p* = 0.0001).

One retrospective study compared PRP to ECSW among athletes and non-athletes [50]. The PRP group received 3 injections, with a total platelet dosage of 5400 × 10^6^. At 2 years follow-up, the PRP group reported statistically decreased VAS scores (*p* < 0.001) in both the athlete and non-athlete subgroups, however, there was no difference in VAS score improvement when compared to those who received ECSW (overall population: *p* = 0.485, athletes: *p* = 0.433, nonathletes: *p* = 0.064).

### Other MSK Conditions

The platelet dosage and efficacy of PRP for treatment of other MSK conditions at 6 months, 1 year, and 2 years post-injection are summarized in Table 4.

**Table 4:** Summary of platelet dosage and efficacy of prp for treatment of other msk conditions at 6 months, 1 year, and 2 years post-injection

Author (Yr)	Comparator	Platelet dose	Efficacy (6 m)	Relative to comparator (6 m)	Efficacy (1y)	Relative to comparator (1y)	Efficacy (2y)	Relative to comparator (2y)
Carpal Tunnel Syndrome								
Uzun et al. (2017) [48]	CSI	3064 × 10^6^	-	-	N/A	N/A	N/A	N/A
**Plantar Fasciitis**								
Soraganvi et al. (2019) [49]	CSI	3000 × 10^6^	+	+	N/A	N/A	N/A	N/A
Srivastava et al. (2022) [50]	CSI	3000 × 10^6^	+	+	N/A	N/A	N/A	N/A
Alessio-Mazzola et al. (2023) [51]	ECSW	5400 × 10^6^	N/A	N/A	N/A	N/A	+	-
**Ankle OCD**								
Akpancar et al. (2019) [52]	Prolotherapy	12,084 × 10^6^	+	-	+	-	N/A	N/A

CSI: Corticosteroid injection, ECSW: Extracorporeal shock wave, N/A: Not applicable

### Carpal Tunnel Syndrome

One prospective cohort study examined the effects of LP-PRP versus corticosteroid injection in treating carpal tunnel syndrome [51]. The platelet dosage used in this study was 3064 × 10^6^, and the injection was done by landmark guidance. At 6 months post-intervention, there was no significant improvement on the Boston Carpal Tunnel Questionnaire (BCTQ) for the PRP group (2.41, SD 0.36; *p* = 0.724 and 1.91, SD 0.18; *p* = 0.601 for the symptom severity and functional status respectively). Additionally, there were no statistical differences in BCTQ scores at 6 months follow-up when compared to the corticosteroid group (*p* = 0.645 and *p* = 0.861 for the symptom severity and functional status respectively).

### Ankle Osteochondral Defect (OCD)

One retrospective cohort study of 49 patients compared 3 injections of PRP for a total dosage of 12,084 × 10^6^ to prolotherapy (PrT) [52]. The average lesion size for these patients was 1.54 cm^2^ in the PRP group and 1.64 cm^2^ in the PrT group respectively. Both groups improved at 21 days, 3 months, 6 months, and 12 months with no difference between the two. The average lesion size was significantly lower in patients with excellent or good outcomes (1.43 ± 0.68 cm^2^ and 1.42 ± 0.63 cm^2^ for PrT and PRP groups, respectively) compared to patients with fair or poor outcomes (2.6 ± 1.21cm^2^ and 2.25 ± 0.2cm^2^ for PrT and PRP groups, respectively).

## Discussion

The practical application of platelet-rich plasma (PRP) research in clinical settings is hampered by considerable variability in preparation methods and reporting standards. With an array of over 50 commercial kits available for PRP production, the resulting products exhibit significant differences in volume, platelet concentration, leukocyte concentration, and growth factor levels [53]. While various classification systems have been proposed to standardize PRP preparation [10, 54, 55], significant heterogeneity persists across the current literature. This review underscores this variability and presents evidence suggesting that dosage might influence clinical outcomes in knee osteoarthritis (OA) and potentially other musculoskeletal conditions.

Platelet dosage emerges as a critical factor influencing PRP efficacy, particularly in knee OA. This review is the first to identify a potential dose-response relationship between platelet quantity and PRP effectiveness for knee OA treatment, pinpointing an optimal threshold of greater than 10 billion platelets for favorable clinical outcomes. Interestingly, this correlation appears more pronounced in patient-reported functional improvements rather than pain alleviation. This aligns with prior literature suggesting that a greater dose may be crucial for long-term success. One study asserted that an absolute count of 10 billion platelets is an important threshold for sustained chondroprotection at 1 year for patients with moderate knee OA, however, these results were not compared to other platelet doses [56]. Another recent study found that a platelet dosage of 5.65 billion produced better improvements in pain and function than a dosage of 2.82 billion at 6 months [57]. Consistent with these observations, our previous review highlighted that studies reporting positive outcomes boasted a mean platelet dose of 5.5 billion, whereas those without significant improvements averaged 2.3 billion [58]. Within the current review, 14 knee OA studies (34.1%) reached a dose of > 10 billion platelets across multiple injections, all achieving positive outcomes at post-injection follow-up.

Some studies suggest better outcomes with multi-injection protocols [21, 26]. For instance, Simental-Mendia et al. observed improved outcomes at 48 weeks with three PRP injections compared to one. Similarly, Subramanyam et al. found superiority of three injections over one or two at one-year follow-up. However, Saqlain et al. found no difference in knee OA outcomes between two PRP injections versus one. Notably, the platelet dosage varied significantly among these studies. A single injection in the study by Saqlain et al. contained more platelets (5.5 billion) than the cumulative dose of three injections by Subramanyam et al. (4.6 billion), indicating that a single, high-dose injection may be equivalent to multiple injections.This prompts inquiries into whether the outcomes of multi-dose protocols depend more on the total platelet dosage rather than the frequency of injections, and whether the cumulative platelet dose from multiple, potentially subtherapeutic injections matches that of a single high-dose injection. A low-dose injection, for example, may not achieve the optimal platelet concentration or volume required for angiogenesis and tissue repair, and therefore, may not be equivalent [8].

Platelet dose response across various musculoskeletal conditions remains uncertain. While positive outcomes were observed in knee OA studies with doses exceeding 10 billion platelets, such findings were limited in other conditions. Some pathologies, like lateral epicondylopathy and plantar fasciitis, showed positive outcomes with lower platelet doses, possibly due to the challenges of injecting high volumes in smaller tendons. Nonetheless, improvements in PRP preparation techniques now facilitate higher doses within smaller volumes, prompting future research to shift focus from concentration-based to total dosage-based assessment.

Beyond knee OA, the research landscape in osteoarthritis (OA) is sparse. Three studies examined PRP for hip OA in this review, with platelet dosages ranging from less than 5 billion to over 10 billion. Treatment efficacy was noted with a minimum platelet dose of 3.5 billion [31], although PRP surpassed comparators only when the dose exceeded 10 billion [30]. Positive outcomes were also reported for glenohumeral OA and carpometacarpal OA at platelet doses below 5 billion [32, 33]. However, due to the limited number of studies on these OA types, drawing definitive conclusions proves challenging, particularly as the study by Kirschner et al. did not show positive outcomes compared to hyaluronic acid.

The impact of platelet dose in tendinopathy is less clear. Our review found fewer studies quantifying platelet dose for specific tendinopathies, complicating generalized recommendations. In addition, the pathogenesis of tendinopathies in various parts of the body may vary, including overuse, degenerative, compressive, neurogenic, and/or inflammatory [59, 60]. Differences in underlying pathophysiology likely impact the efficacy of PRP, especially since both leukocyte-rich and leukocyte-poor preparations are used. Hence, ideal platelet dosing may vary across different tendinopathies.

In rotator cuff tendinopathy platelet doses ranged from less than 5 billion to almost 16 billion across five analyzed studies. In the study that reached 16 billion platelets [39], patients had improved pain and functional scores compared to the control. However, it required 4 injections to reach this number, which again may emphasize the impact of the platelet dose itself instead of the number of injections. Further studies should aim to compare similar platelet doses in both single- and multi–injection protocols as well as assess whether multiple injections of lower dosage are comparable to a single injection of a higher dose. In addition, all but one study injected the PRP into the subacromial space as opposed to the tendon itself. The impact of peri tendinous PRP has not been well studied, and the contribution of the subacromial bursa to pain and dysfunction related to shoulder impingement and rotator cuff tendinopathy is still debated [61].

Among lateral epicondylopathy studies, one had a platelet dose below 5 billion [35], while two ranged from 5 to 10 billion [34, 36]. All three reported significant pain improvement. For gluteal tendinopathy, patient-reported outcomes improved with a 5–10 billion platelet dosage [42, 43], although one study found no difference compared to saline placebo, possibly due to the lack of ultrasound guidance. In patellar tendinopathy, both less than 5 billion and 5–10 billion platelet doses showed pain and function improvement, though the study with less than 5 billion platelets found no difference compared to normal saline, indicating a likely placebo effect [44, 45]. Similarly, Achilles tendinopathy studies reported symptom improvement with less than 5 billion platelets [46, 47]. All three plantar fasciitis studies yielded positive outcomes post-injection. Two had platelet doses below 5 billion [48, 49], while one fell between 5 and 10 billion [50]. However, apart from one rotator cuff tendinopathy study [39], no tendinopathy trials exceeded 10 billion platelets, making it challenging to ascertain whether a dose surpassing 10 billion is necessary for clinical improvement in tendinopathic pathologies.

The optimal platelet dosage for musculoskeletal conditions beyond OA and tendinopathy remains uncertain. In the sole ankle osteochondral defect study, patient outcomes improved with a platelet dosage exceeding 10 billion [52]. However, in the carpal tunnel syndrome study, a platelet dose below 5 billion failed to improve patient outcomes [51].

### Limitations

Our review presents several limitations. Firstly, our inclusion of only English-published studies introduces a potential for selection bias, although prior research suggests a low risk of bias [62]. Secondly, over half of the studies identified for full-text review were excluded due to insufficient reporting of platelet dosage. This decision aligns with the 2017 Minimum Information for Studies Evaluating Biologics in Orthopaedics, which mandates cell count reporting as the standard. Consequently, our meta-analysis primarily focused on knee OA, given its prevalence in the literature, thereby hindering the analysis of other pathologies. Quantitative analysis of the remaining musculoskeletal conditions was impeded by limited study numbers per condition and heterogeneity across studies. The studies also displayed a high level of heterogeneity amongst control groups, making generalization more difficult. Publication bias is a concern, as potentially effective studies may be overrepresented. Prior research indicates inflated effect sizes due to selective publication in knee OA, hip OA, and rotator cuff tendinopathy [63–65]. Around one-third of the studies in our review were prospective or retrospective cohort studies. While the included randomized controlled trials (RCTs) demonstrated an overall low risk of bias, non-RCTs were not subject to bias assessment, potentially influencing our analysis. This may even include the risk of industry bias, which was not assessed in all studies. Lastly, our analysis did not consider variations in leukocyte concentration of PRP, image guidance during PRP administration, or the minimal clinically important difference (MCID) for the different musculoskeletal conditions studied.

## Conclusion

PRP can provide therapeutic benefits in patients with chronic musculoskeletal conditions including osteoarthritis and tendinopathy. There is a potential dose-response relationship between platelet dose and PRP effectiveness with an optimal dosage of greater than 10 billion to achieve maximum results for knee OA. The heterogeneity and lack of PRP reporting and standardization in the current literature limits recommendations on other musculoskeletal conditions. Further research is warranted to elucidate optimal platelet dosages.

## Key References

- Murray IR, Geeslin AG, Goudie EB, Petrigliano FA, LaPrade RF. Minimum Information for Studies Evaluating Biologics in Orthopaedics (MIBO): platelet-rich plasma and mesenchymal stem cells. J Bone Joint Surg Am. 2017;99(10):809–19. The paper establishes expert consensus on the minimum reporting requirements for clinical studies evaluating PRP and MSCs.
- Everts PA, Lana JF, Onishi K, Buford D, Peng J, Mahmood A et al. Angiogenesis and Tissue Repair Depend on Platelet Dosing and Bioformulation Strategies Following Orthobiological Platelet-Rich Plasma Procedures: A Narrative Review. Biomedicines. 2023;11(7):1922. The manuscript highlights the pathophysology and clinical importance of platelet dosing for PRP procedures.
- Patel S, Gahlaut S, Thami T, Chouhan DK, Jain A, Dhillon MS. Comparison of Conventional Dose Versus Superdose Platelet-Rich Plasma for Knee Osteoarthritis: A Prospective, Triple-Blind, Randomized Clinical Trial. Orthop J Sports Med. 2024;12(2):23259671241227863. A Randomized clinical trial demonstrating that a higher dose PRP preparation outperforms a lower dose PRP.
- Berrigan WA, Bailowitz Z, Park A, Reddy A, Liu R, Lansdown D. A Greater platelet dose May yield better clinical outcomes for Platelet-Rich Plasma in the treatment of knee osteoarthritis: a systematic review. Arthroscopy. 2024. A systematic review showing that negative PRP studies used a lower dose PRP than positive PRP studies.

## Electronic Supplementary Material

Below is the link to the electronic supplementary material.

Supplementary Material 1

## Data Availability

No datasets were generated or analysed during the current study.

## References

[1] Bacevich BM, Smith RDJ, Reihl AM, Mazzocca AD, Hutchinson ID. Advances with platelet-rich plasma for Bone Healing. Biologics. 2024;18:29–59. 38299120 10.2147/BTT.S290341 PMC10827634

[2] Nelson PA, George T, Bowen E, Sheean AJ, Bedi A. An update on Orthobiologics: cautious optimism. Am J Sports Med. 2024;52(1):242–57. 38164688 10.1177/03635465231192473

[3] Marx RE. Platelet-rich plasma (PRP): what is PRP and what is not PRP? Implant Dent. 2001;10(4):225–8. 11813662 10.1097/00008505-200110000-00002

[4] Murray IR, Geeslin AG, Goudie EB, Petrigliano FA, LaPrade RF. Minimum Information for Studies Evaluating Biologics in Orthopaedics (MIBO): platelet-rich plasma and mesenchymal stem cells. J Bone Joint Surg Am. 2017;99(10):809–19. 28509821 10.2106/JBJS.16.00793

[5] Southworth TM, Naveen NB, Tauro TM, Leong NL, Cole BJ. The use of platelet-rich plasma in symptomatic knee osteoarthritis. J Knee Surg. 2019;32(1):37–45. 30423591 10.1055/s-0038-1675170

[6] Townsend C, Von Rickenbach KJ, Bailowitz Z, Gellhorn AC. Post-procedure Protocols following platelet-rich plasma injections for Tendinopathy: a systematic review. Pm r. 2020;12(9):904–15. 32103599 10.1002/pmrj.12347

[7] Everts PA, van Erp A, DeSimone A, Cohen DS, Gardner RD. Platelet Rich plasma in Orthopedic Surgical Medicine. Platelets. 2021;32(2):163–74. 33400591 10.1080/09537104.2020.1869717

[8] Everts PA, Lana JF, Onishi K, Buford D, Peng J, Mahmood A, et al. Angiogenesis and tissue repair depend on platelet dosing and Bioformulation Strategies following Orthobiological platelet-rich plasma procedures: a narrative review. Biomedicines. 2023;11(7):1922. 37509560 10.3390/biomedicines11071922 PMC10377284

[9] Mathes T, Pieper D. Clarifying the distinction between case series and cohort studies in systematic reviews of comparative studies: potential impact on body of evidence and workload. BMC Med Res Methodol. 2017;17(1):107. 28716005 10.1186/s12874-017-0391-8 PMC5513097

[10] Lana J, Purita J, Paulus C, Huber SC, Rodrigues BL, Rodrigues AA, et al. Contributions for classification of platelet rich plasma - proposal of a new classification: MARSPILL. Regen Med. 2017;12(5):565–74. 28758836 10.2217/rme-2017-0042

[11] Higgins JP, Altman DG, Gøtzsche PC, Jüni P, Moher D, Oxman AD, et al. The Cochrane collaboration’s tool for assessing risk of bias in randomised trials. BMJ. 2011;343:d5928. 22008217 10.1136/bmj.d5928 PMC3196245

[12] Montañez-Heredia E, Irízar S, Huertas PJ, Otero E, Del Valle M, Prat I et al. Intra-articular injections of platelet-rich plasma versus hyaluronic acid in the treatment of osteoarthritic knee pain: A randomized clinical trial in the context of the Spanish national health care system. International Journal of Molecular Sciences. 2016;17(7). 10.3390/ijms17071064 PMC4964440 27384560

[13] Sandhu JS, Sagar S, Sahu S, Raghuvanshi R, Paliwal H, Jain BK. A prospective study of intra- articular injection of platelet Rich plasma (PRP) in knee osteoarthritis. Eur J Mol Clin Med. 2022;9(3):6014–22.

[14] Saqlain L, Hussain SS, Keerio NH, Qureshi MA, Valecha NK, Noor SS. Effect of platelet Rich plasma injection effect on knee osteoarthritis in Elderly: Single Dose versus Double Dose Randomized Clinical Trial.84 – 9.

[15] Silvestre A, Lintingre PF, Pesquer L, Meyer P, Moreau-Durieux MH, Dallaudiére B. Retrospective analysis of responders and impaired patients with knee osteoarthritis treated with two consecutive injections of very pure platelet-rich plasma (PRP). Bioeng (Basel). 2023;10(8). 10.3390/bioengineering10080922 PMC10451974 37627807

[16] Zaffagnini S, Andriolo L, Boffa A, Poggi A, Cenacchi A, Busacca M, et al. Microfragmented adipose tissue Versus platelet-rich plasma for the treatment of knee osteoarthritis: a prospective Randomized Controlled Trial at 2-Year follow-up. Am J Sports Med. 2022;50(11):2881–92. 35984721 10.1177/03635465221115821

[17] Li M, Huang Z, Wang S, Di Z, Zhang J, Liu H. Intra-articular injections of platelet-rich plasma vs. hyaluronic acid in patients with knee osteoarthritis: preliminary follow-up results at 6-months. Experimental Therapeutic Med. 2021;21(6). 10.3892/etm.2021.10030 PMC8056115 33884036

[18] Saita Y, Kobayashi Y, Nishio H, Wakayama T, Fukusato S, Uchino S, et al. Predictors of effectiveness of platelet-rich plasma therapy for knee osteoarthritis: a retrospective cohort study. J Clin Med. 2021;10:19. 10.3390/jcm10194514 PMC8509123 34640529

[19] Sánchez M, Jorquera C, de Dicastillo LL, Fiz N, Knörr J, Beitia M et al. Real-world evidence to assess the effectiveness of platelet-rich plasma in the treatment of knee degenerative pathology: a prospective observational study. 10.1177/1759720X221100304 PMC9201351 35721321

[20] Taniguchi Y, Yoshioka T, Kanamori A, Aoto K, Sugaya H, Yamazaki M. Intra-articular platelet-rich plasma (PRP) injections for treating knee pain associated with osteoarthritis of the knee in the Japanese population: a phase I and IIa clinical trial. Nagoya J Med Sci. 2018;80(1):39–51. 29581613 10.18999/nagjms.80.1.39 PMC5857500

[21] Simental-Mendía M, Acosta-Olivo CA, Hernández-Rodríguez AN, Santos-Santos OR, de la Garza-Castro S, Peña-Martínez VM, et al. Intraarticular injection of platelet-rich plasma in knee osteoarthritis: single versus triple application approach. Pilot study. Acta Reumatol Port. 2019;44(2):138–44. 31243258

[22] Akan O, Sarikaya NO, Kocyigit H. Efficacy of platelet-rich plasma administration in patients with severe knee osteoarthritis: can platelet-rich plasma administration delay arthroplasty in this patient population?9473–83.

[23] Bec C, Rousset A, Brandin T, François P, Rabarimeriarijaona S, Dumoulin C et al. A retrospective analysis of characteristic features of responders and impaired patients to a single injection of pure platelet-rich plasma in knee osteoarthritis. J Clin Med. 2021;10(8). 10.3390/jcm10081748 PMC8073986 33920633

[24] Govila VK, Sanghi S, Singh V, Kumar A. PLATELET-RICH PLASMA (PRP) THERAPY FOR KNEE ARTHRITIS IN A TERTIARY CARE TEACHING HOSPITAL STUDY.72 – 5.

[25] Guillibert C, Charpin C, Raffray M, Benmenni A, Dehaut FX, El Ghobeira G et al. Single injection of high volume of autologous pure PRP provides a significant improvement in knee osteoarthritis: a prospective Routine Care Study. Int J Mol Sci. 2019;20(6). 10.3390/ijms20061327 PMC6472196 30884774

[26] Subramanyam K, Alguvelly R, Mundargi A, Khanchandani P. Single versus multi-dose intra-articular injection of platelet rich plasma in early stages of osteoarthritis of the knee: a single-blind, randomized, superiority trial. Archives Rheumatol. 2021;36(3):326–34. 10.46497/ArchRheumatol.2021.8408 PMC8612497 34870163

[27] Tucker JD, Goetz LL, Duncan MB, Gilman JB, Elmore LW, Sell SA, et al. Randomized, Placebo-controlled analysis of the knee synovial environment following platelet-rich plasma treatment for knee osteoarthritis. PM R. 2021;13(7):707–19. 33492733 10.1002/pmrj.12561

[28] Palco M, Fenga D, Basile GC, Rizzo P, Cavalieri B, Leonetti D et al. Platelet-Rich plasma combined with hyaluronic acid versus leucocyte and platelet-rich plasma in the conservative treatment of knee osteoarthritis. A retrospective study. Medicina (Kaunas. Lithuania). 2021;57(3). 10.3390/medicina57030232 PMC7998747 33802325

[29] Wang YC, Lee CL, Chen YJ, Tien YC, Lin SY, Chen CH et al. Comparing the efficacy of Intra-articular single platelet-rich plasma(PRP) versus Novel Crosslinked Hyaluronic Acid for early-stage knee osteoarthritis: a prospective, Double-Blind, randomized controlled trial. Med (Kaunas). 2022;58(8). 10.3390/medicina58081028 PMC9415551 36013495

[30] Nouri F, Babaee M, Peydayesh P, Esmaily H, Raeissadat SA. Comparison between the effects of ultrasound guided intra-articular injections of platelet-rich plasma (PRP), high molecular weight hyaluronic acid, and their combination in hip osteoarthritis: a randomized clinical trial. BMC Musculoskelet Disord. 2022;23(1):856. 36096771 10.1186/s12891-022-05787-8 PMC9464606

[31] Villanova-López MM, Núñez-Núñez M, Fernández-Prieto D, González-López C, García-Donaire J, Pérez-Pérez A et al. Randomized, double-blind, controlled trial, phase III, to evaluate the use of platelet-rich plasma versus hyaluronic acid in hip coxarthrosis.134-42. 10.1016/j.recot.2019.09.008 31902736

[32] Kirschner JS, Cheng J, Creighton A, Dundas M, Beatty NR, Kingsbury D et al. Efficacy of ultrasound-guided glenohumeral joint injections of platelet-rich plasma versus hyaluronic acid in the treatment of glenohumeral osteoarthritis: a randomized, double-blind controlled trial.S6. 10.1097/JSM.0000000000001029 PMC9481749 35316820

[33] Hasley IB, Bies MM, Hollman JH, Carta KG, Sellon JL, Brault JS. Platelet-Rich Plasma Injection for Thumb Carpometacarpal Joint Osteoarthritis. 10.1016/j.arrct.2023.100257 PMC10036221 36968169

[34] Lim W, Park SH, Kim B, Kang SW, Lee JW, Moon YL. Relationship of cytokine levels and clinical effect on platelet-rich plasma-treated lateral epicondylitis.913 – 20. 10.1002/jor.23714 28851099

[35] Gupta PK, Acharya A, Khanna V, Roy S, Khillan K, Sambandam SN. PRP versus steroids in a deadlock for efficacy: long-term stability versus short-term intensity-results from a randomised trial.285 – 94. 10.1007/s12306-019-00619-w 31448392

[36] Alessio-Mazzola M, Repetto I, Biti B, Trentini R, Formica M, Felli L. Autologous US-guided PRP injection versus US-guided focal extracorporeal shock wave therapy for chronic lateral epicondylitis: a minimum of 2-year follow-up retrospective comparative study.2309499017749986. 10.1177/2309499017749986 29320964

[37] Rossi LA, Piuzzi N, Giunta D, Tanoira I, Brandariz R, Pasqualini I et al. Subacromial platelet-rich plasma injections decrease Pain and improve functional outcomes in patients with refractory rotator cuff Tendinopathy.2745-53. 10.1016/j.arthro.2021.03.079 33892072

[38] Nejati P, Ghahremaninia A, Naderi F, Gharibzadeh S, Mazaherinezhad A. Treatment of subacromial impingement syndrome: platelet-rich plasma or exercise therapy? A randomized controlled trial. 10.1177/2325967117702366 PMC5439655 28567426

[39] Cai Y, Sun ZX, Liao BK, Song ZQ, Xiao T, Zhu PF. Sodium Hyaluronate and Platelet-Rich Plasma for Partial-Thickness Rotator Cuff Tears.227 – 33. 10.1249/MSS.0000000000001781 PMC6336488 30199423

[40] Jo CH, Lee SY, Yoon KS, Oh S, Shin S. Allogeneic platelet-rich plasma Versus Corticosteroid Injection for the treatment of Rotator Cuff Disease: a randomized controlled Trial.2129-37. 10.2106/JBJS.19.01411 33044249

[41] Hewavithana PB, Wettasinghe MC, Hettiarachchi G, Ratnayaka M, Suraweera H, Wickramasinghe ND et al. Effectiveness of single intra-bursal injection of platelet-rich plasma against corticosteroid under ultrasonography guidance for shoulder impingement syndrome: a randomized clinical trial. 10.1007/s00256-023-04373-w 37266723

[42] Fitzpatrick J, Bulsara MK, O’Donnell J, McCrory PR, Zheng MH. The effectiveness of platelet-rich plasma injections in gluteal tendinopathy: a Randomized, double-blind controlled trial comparing a single platelet-rich plasma injection with a single corticosteroid Injection.933-9. 10.1177/0363546517745525 29293361

[43] Thompson G, Pearson JF. No attributable effects of PRP on greater trochanteric pain syndrome.22–32. 31830014

[44] Scott A, Laprade R, Harmon K, Filardo G, Kon E, Villa SD et al. Platelet-rich plasma (PRP) for patellar tendinopathy: a randomized controlled trial of leukocyte-rich PRP or leukocyte-poor PRP vs. saline.S26. 10.1177/0363546519837954 31038979

[45] Rodas G, Soler-Rich R, Rius-Tarruella J, Alomar X, Balius R, Orozco L et al. Effect of Autologous Expanded Bone Marrow Mesenchymal Stem Cells or Leukocyte-Poor Platelet-Rich Plasma in Chronic Patellar Tendinopathy (With Gap > 3 mm): Preliminary Outcomes After 6 Months of a Double-Blind, Randomized, Prospective Study.1492 – 504. 10.1177/0363546521998725 33783227

[46] Erroi D, Sigona M, Suarez T, Trischitta D, Pavan A, Vulpiani MC et al. Conservative treatment for Insertional Achilles Tendinopathy: platelet-rich plasma and focused shock waves. A retrospective study.98–106. 10.11138/mltj/2017.7.1.098 PMC5505601 28717617

[47] Usuelli FG, Grassi M, Maccario C, Vigano M, Lanfranchi L, Alfieri Montrasio U, et al. Intratendinous adipose-derived stromal vascular fraction (SVF) injection provides a safe, efficacious treatment for Achilles tendinopathy: results of a randomized controlled clinical trial at a 6-month follow-up. Knee Surg Sports Traumatol Arthrosc. 2018;26(7):2000–10. 28251260 10.1007/s00167-017-4479-9

[48] Soraganvi P, Nagakiran KV, Raghavendra-Raju RP, Anilkumar D, Wooly S, Basti BD et al. Is platelet-rich plasma injection more effective than steroid injection in the treatment of chronic plantar fasciitis in achieving long-term relief?8–14. 10.5704/MOJ.1911.002 PMC6915312 31890104

[49] Srivastava V, Vishwas, Rathi R, Ln M, Bl K. PLANTAR FASCIITIS TREATMENT WITH PLATELET-RICH PLASMA INJECTION VERSUS STEROID INJECTION.120-2.

[50] Alessio-Mazzola M, Stambazzi C, Ursino C, Tagliafico A, Trentini R, Formica M. Ultrasound-guided autologous platelet-rich plasma injections Versus Focal Ultrasound-guided extracorporeal shockwave therapy for Plantar fasciitis in athletes and nonathletes: a retrospective comparative study with Minimum 2-Year Follow-Up.417 – 21. 10.1053/j.jfas.2022.10.005 36396549

[51] Uzun H, Bitik O, Uzun Ö, Ersoy US, Aktaş E. Platelet-rich plasma versus corticosteroid injections for carpal tunnel syndrome.301-5. 10.1080/2000656X.2016.1260025 27921443

[52] Akpancar S, Gül D. Comparison of platelet rich plasma and prolotherapy in the management of osteochondral lesions of the talus: a retrospective cohort study.5640-7. 10.12659/MSM.914111 PMC6685325 31358724

[53] Oudelaar BW, Peerbooms JC, Huis in ‘t Veld R, Vochteloo AJH. Concentrations of Blood Components in Commercial Platelet-Rich Plasma Separation Systems: A Review of the Literature. The American Journal of Sports Medicine. 2019;47(2):479 – 87. 10.1177/0363546517746112 29337592

[54] Magalon J, Brandin T, Francois P, Degioanni C, De Maria L, Grimaud F, et al. Technical and biological review of authorized medical devices for platelets-rich plasma preparation in the field of regenerative medicine. Platelets. 2021;32(2):200–8. 33155867 10.1080/09537104.2020.1832653

[55] Mautner K, Malanga GA, Smith J, Shiple B, Ibrahim V, Sampson S, et al. A call for a standard classification system for future biologic research: the rationale for new PRP nomenclature. Pm r. 2015;7(4 Suppl):S53–9. 25864661 10.1016/j.pmrj.2015.02.005

[56] Bansal H, Leon J, Pont JL, Wilson DA, Bansal A, Agarwal D, et al. Platelet-rich plasma (PRP) in osteoarthritis (OA) knee: correct dose critical for long term clinical efficacy. Sci Rep. 2021;11(1):3971. 33597586 10.1038/s41598-021-83025-2 PMC7889864

[57] Patel S, Gahlaut S, Thami T, Chouhan DK, Jain A, Dhillon MS. Comparison of Conventional Dose Versus Superdose platelet-rich plasma for knee osteoarthritis: a prospective, Triple-Blind, randomized clinical trial. Orthop J Sports Med. 2024;12(2):23259671241227863. 38410168 10.1177/23259671241227863 PMC10896053

[58] Berrigan WA, Bailowitz Z, Park A, Reddy A, Liu R, Lansdown D. A Greater Platelet Dose May Yield Better Clinical Outcomes for Platelet-Rich Plasma in the Treatment of Knee Osteoarthritis: A Systematic Review. Arthroscopy. 2024. 10.1016/j.arthro.2024.03.018 38513880

[59] Ackermann PW, Alim MA, Pejler G, Peterson M. Tendon pain – what are the mechanisms behind it? Scandinavian J Pain. 2023;23(1):14–24. 10.1515/sjpain-2022-0018 35850720

[60] Wasker SVZ, Challoumas D, Weng W, Murrell GAC, Millar NL. Is neurogenic inflammation involved in tendinopathy? A systematic review. BMJ Open Sport Exerc Med. 2023;9(1):e001494. 36793930 10.1136/bmjsem-2022-001494 PMC9923261

[61] Klatte-Schulz F, Thiele K, Scheibel M, Duda GN, Wildemann B. Subacromial Bursa: a neglected tissue is gaining more and more attention in clinical and experimental research. Cells. 2022;11(4). 10.3390/cells11040663 PMC8870132 35203311

[62] Morrison LJ, Eby D, Veigas PV, Zhan C, Kiss A, Arcieri V, et al. Implementation trial of the basic life support termination of resuscitation rule: reducing the transport of futile out-of-hospital cardiac arrests. Resuscitation. 2014;85(4):486–91. 24361458 10.1016/j.resuscitation.2013.12.013

[63] Nie LY, Zhao K, Ruan J, Xue J. Effectiveness of Platelet-Rich Plasma in the treatment of knee osteoarthritis: a Meta-analysis of Randomized Controlled clinical trials. Orthop J Sports Med. 2021;9(3):2325967120973284. 33718505 10.1177/2325967120973284 PMC7930657

[64] Kim JH, Park YB, Ha CW. Are leukocyte-poor or multiple injections of platelet-rich plasma more effective than hyaluronic acid for knee osteoarthritis? A systematic review and meta-analysis of randomized controlled trials. Archives of Orthopaedic and Trauma Surgery; 2022. 10.1007/s00402-022-04637-5 36173473

[65] Chen CPC, Chen JL, Hsu CC, Pei YC, Chang WH, Lu HC. Injecting autologous platelet rich plasma solely into the knee joint is not adequate in treating geriatric patients with moderate to severe knee osteoarthritis.1–6. 10.1016/j.exger.2019.01.018 30664923

