# Supplementary material for: The Effect of Platelet Dose on Outcomes after Platelet Rich Plasma Injections for Musculoskeletal Conditions: A Systematic Review and Meta-Analysis
Source: Curr Rev Musculoskelet Med. 2024 Sep 27;17(12):570–88. doi: 10.1007/s12178-024-09922-x (PMC11652557; doi:10.1007/s12178-024-09922-x)

**Appendix 1:** PRP Search Appendix

| DATABASE | SEARCH STRATEGY |
| --- | --- |
|  |  |
| PubMed | (“platelet-rich plasma” OR "Platelet-Rich Plasma"[Mesh] OR PRP) AND (joint OR "Joints"[Mesh] OR arthritis OR "Arthritis"[Mesh] OR osteoarthritis OR “degenerative arthritis” OR “chondral lesion” OR tendon OR "Tendons"[Mesh] OR tendinopathy OR "Tendinopathy"[Mesh] OR tendinosis OR tendinitis OR “tendon tear” OR ligament OR "Ligaments"[Mesh] OR “ligament tear” OR muscle OR "Muscles"[Mesh] OR meniscus OR "Meniscus"[Mesh] OR labrum OR fascia OR "Fascia"[Mesh] OR bone OR "Bone and Bones"[Mesh] OR fracture OR "Fractures, Bone"[Mesh] OR “peripheral nerves” OR "Peripheral Nerves"[Mesh]) AND ("Randomized Controlled Trial" [Publication Type] OR “randomized controlled trial” OR RCT OR “randomized trial” OR “randomised controlled trial” OR “randomised trial” OR “clinical trial” OR "Clinical Trial" [Publication Type] OR "Controlled Clinical Trial" [Publication Type] OR "Pragmatic Clinical Trial" [Publication Type] OR “cohort study” OR "Cohort Studies"[Mesh] OR “prospective study” OR "Prospective Studies"[Mesh] OR “retrospective study” OR "Retrospective Studies"[Mesh]) |
| Web of Science | (“platelet-rich plasma” OR PRP) AND (joint OR Joints OR arthritis OR osteoarthritis OR “degenerative arthritis” OR “chondral lesion” OR tendon OR Tendons OR tendinopathy OR tendinosis OR tendinitis OR “tendon tear” OR ligament OR Ligaments OR “ligament tear” OR muscle OR muscles OR meniscus OR labrum OR fascia OR bone OR Bones OR fracture OR Fractures OR “peripheral nerves”) AND (“randomized controlled trial” OR RCT OR “randomized trial” OR “randomised controlled trial” OR “randomised trial” OR “clinical trial” OR "Controlled Clinical Trial" OR "Pragmatic Clinical Trial" OR “cohort study” OR "Cohort Studies" OR “prospective study” OR "Prospective Studies" OR “retrospective study” OR "Retrospective Studies") |
| Embase | ('platelet-rich plasma'/exp OR 'platelet-rich plasma' OR 'prp'/exp OR prp) AND ('joint'/exp OR joint OR 'joints'/exp OR joints OR 'arthritis'/exp OR arthritis OR 'osteoarthritis'/exp OR osteoarthritis OR 'degenerative arthritis'/exp OR 'degenerative arthritis' OR 'chondral lesion'/exp OR 'chondral lesion' OR 'tendon'/exp OR tendon OR 'tendons'/exp OR tendons OR 'tendinopathy'/exp OR tendinopathy OR 'tendinosis'/exp OR tendinosis OR 'tendinitis'/exp OR tendinitis OR 'tendon tear'/exp OR 'tendon tear' OR 'ligament'/exp OR ligament OR 'ligaments'/exp OR ligaments OR 'ligament tear' OR 'muscle'/exp OR muscle OR 'muscles'/exp OR muscles OR 'meniscus'/exp OR meniscus OR 'labrum'/exp OR labrum OR 'fascia'/exp OR fascia OR 'bone'/exp OR bone OR 'bones'/exp OR bones OR 'fracture'/exp OR fracture OR 'fractures'/exp OR fractures OR 'peripheral nerves'/exp OR 'peripheral nerves') AND ('randomized controlled trial'/exp OR 'randomized controlled trial' OR rct OR 'randomized trial' OR 'randomisedcontrolled trial'/exp OR 'randomised controlled trial' OR 'randomised trial' OR 'clinical trial'/exp OR 'clinical trial' OR 'controlled clinical trial'/exp OR 'controlled clinical trial' OR 'pragmatic clinical trial'/exp OR 'pragmatic clinical trial' OR 'cohort study'/exp OR 'cohort study' OR 'cohort studies'/exp OR 'cohort studies' OR 'prospective study'/exp OR 'prospective study' OR 'prospective studies'/exp OR 'prospective studies' OR 'retrospective study'/exp OR 'retrospective study' OR 'retrospective studies'/exp OR 'retrospective studies') |
| Cochrane Library | (“platelet-rich plasma” OR PRP) AND (joint OR Joints OR arthritis OR osteoarthritis OR “degenerative arthritis” OR “chondral lesion” OR tendon OR Tendons OR tendinopathy OR tendinosis OR tendinitis OR “tendon tear” OR ligament OR Ligaments OR “ligament tear” OR muscle OR muscles OR meniscus OR labrum OR fascia OR bone OR Bones OR fracture OR Fractures OR “peripheral nerves”) AND (“randomized controlled trial” OR RCT OR “randomized trial” OR “randomised controlled trial” OR “randomised trial” OR “clinical trial” OR "Controlled Clinical Trial" OR "Pragmatic Clinical Trial" OR “cohort study” OR "Cohort Studies" OR “prospective study” OR "Prospective Studies" OR “retrospective study” OR "Retrospective Studies") |

**Appendix 2:** Regression Coefficients

WOMAC – 6 months


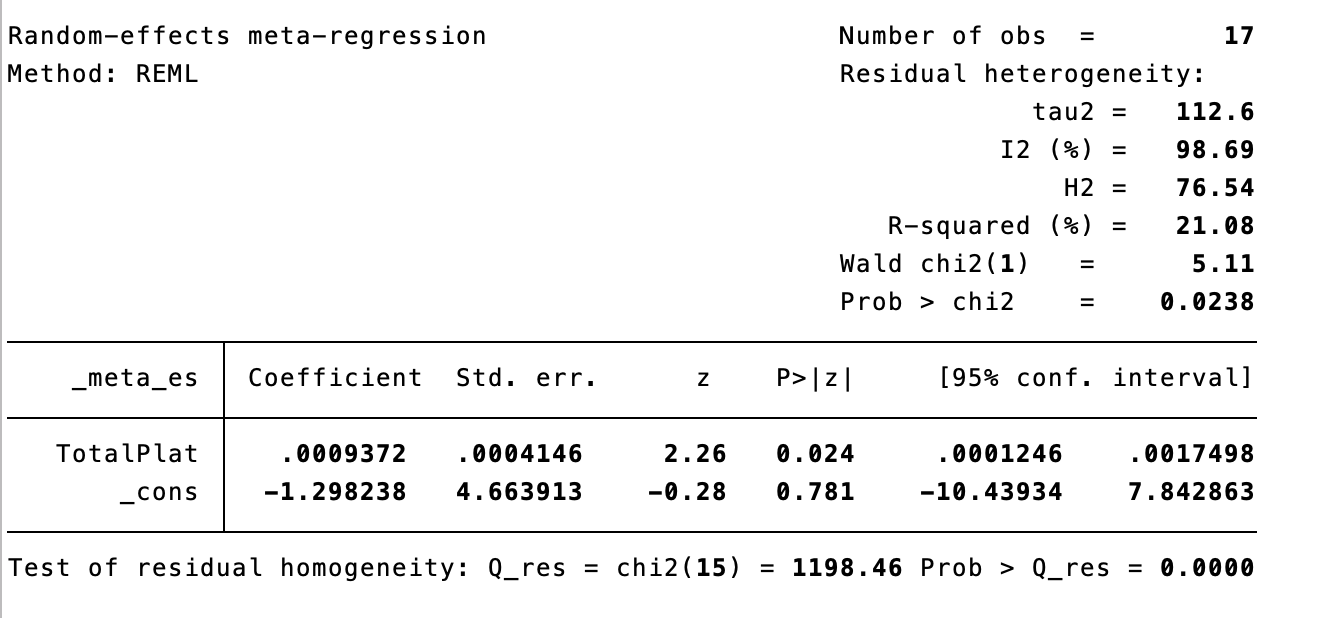


WOMAC – 12 months


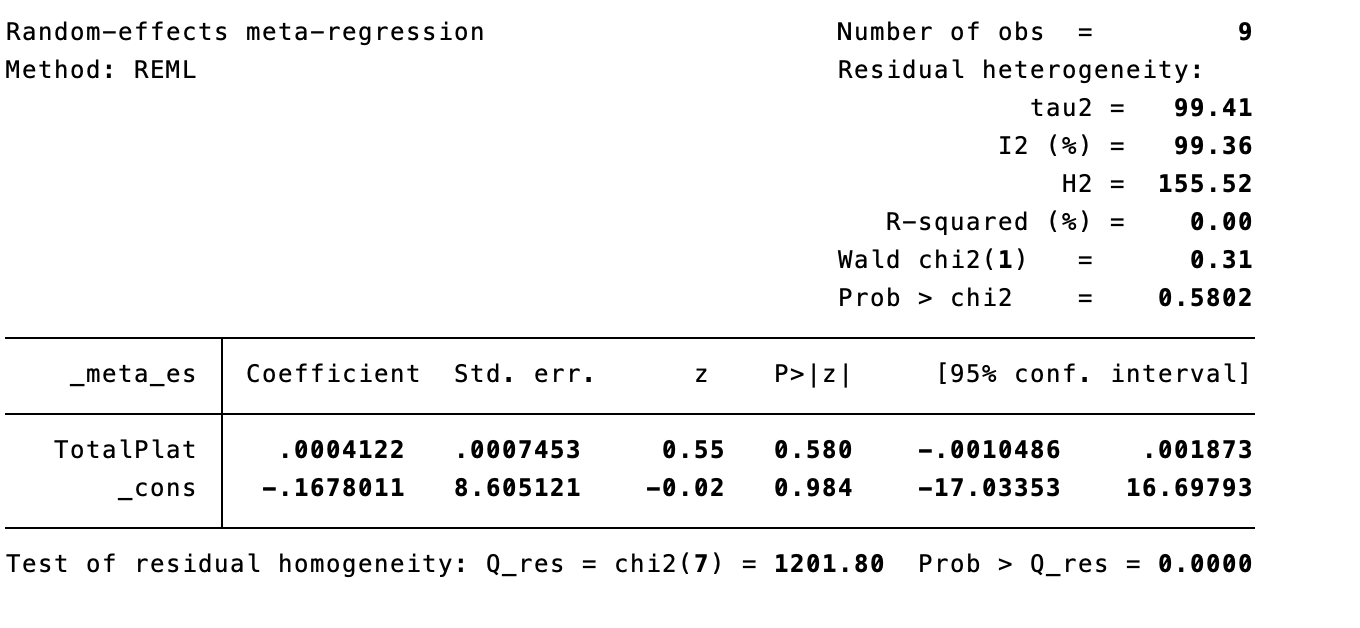


VAS – 6 months


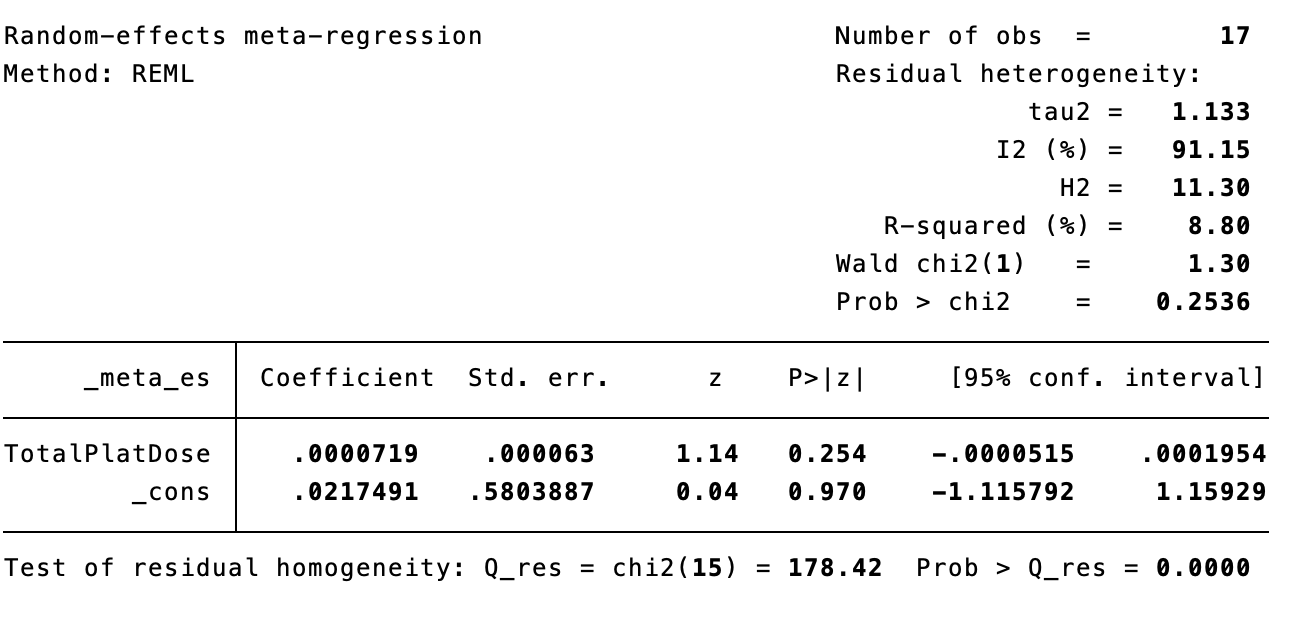


VAS – 12 months


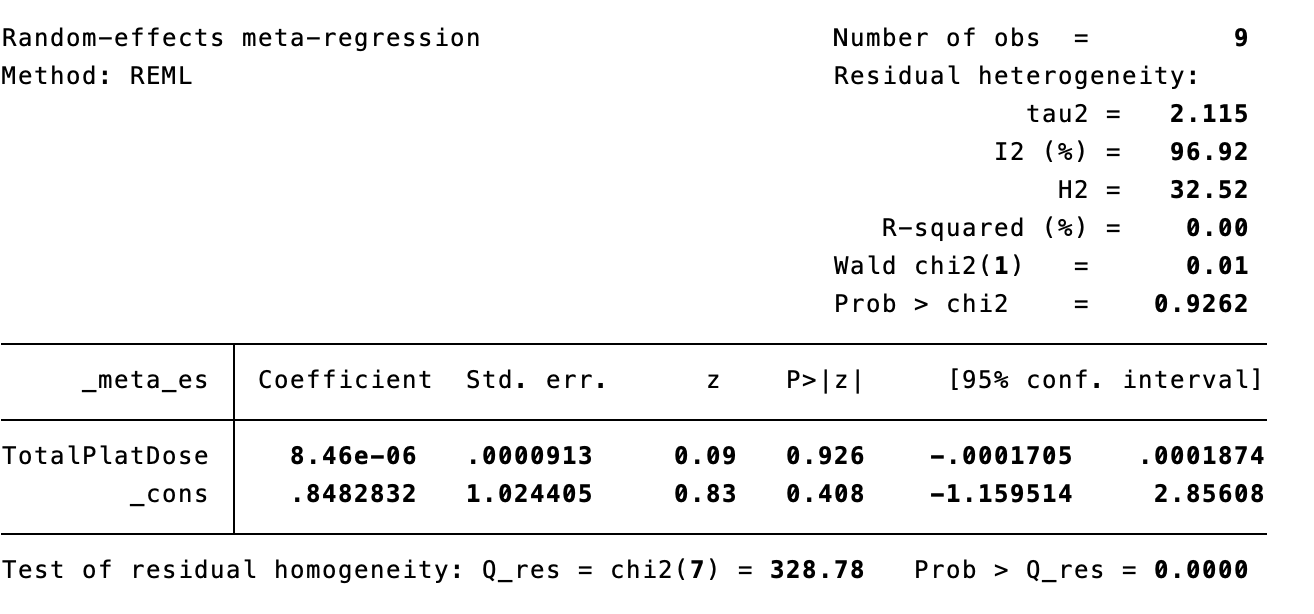


IKDC – 6 months
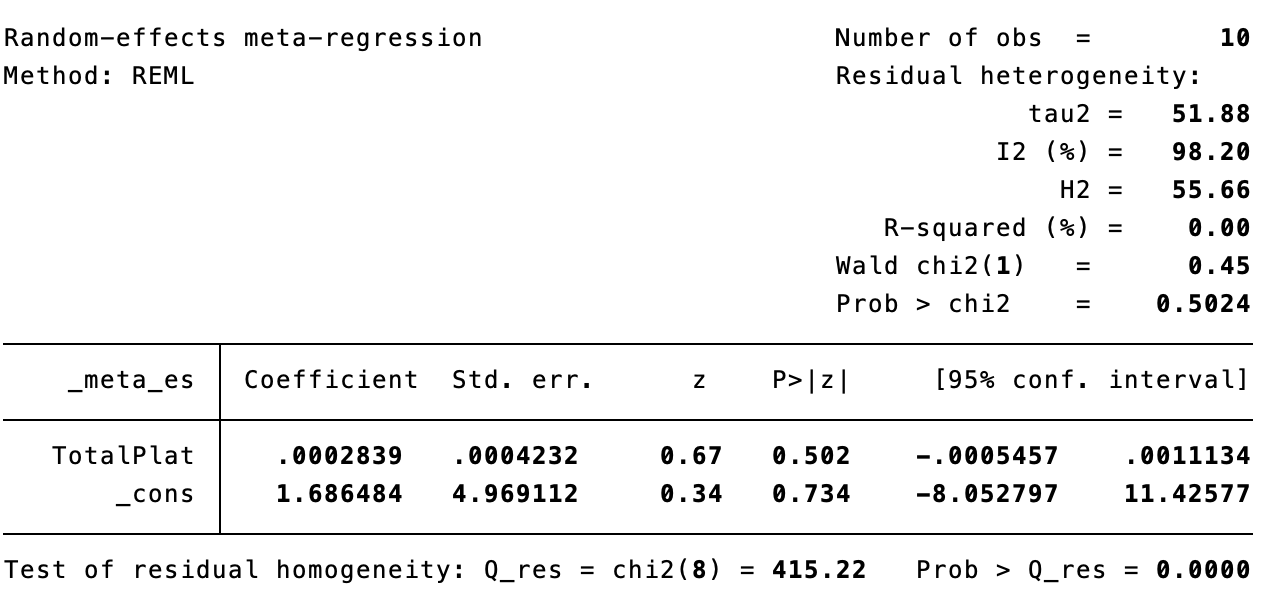


IKDC – 12 months


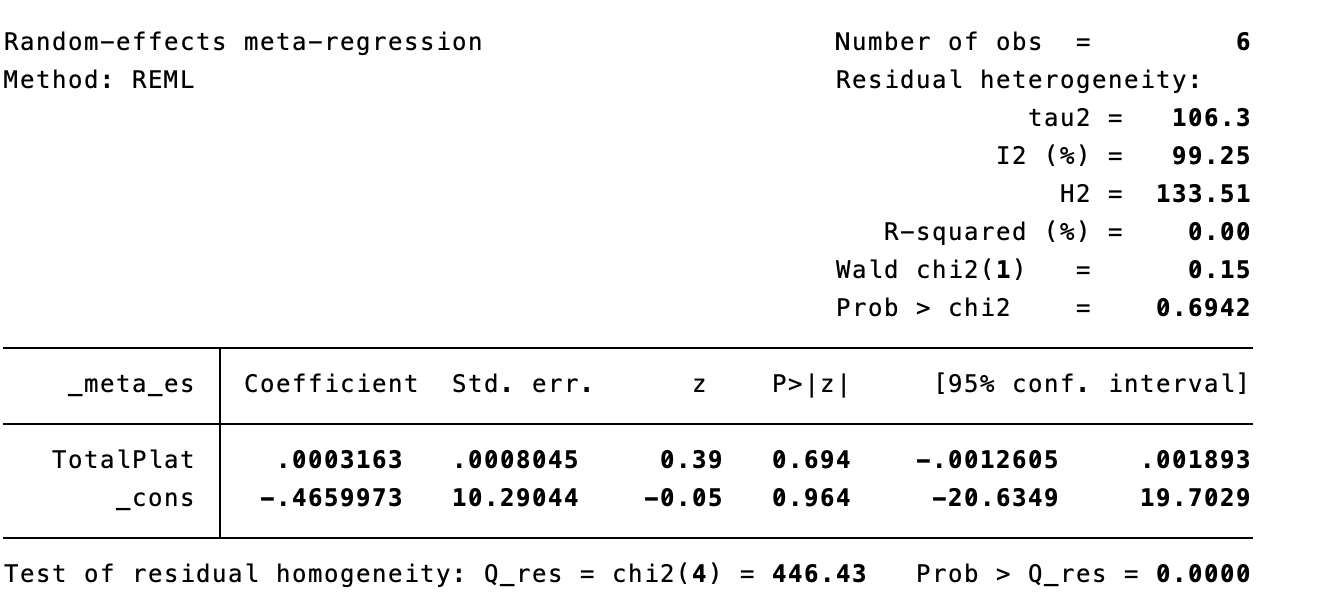


KOOS Sport – 6 months


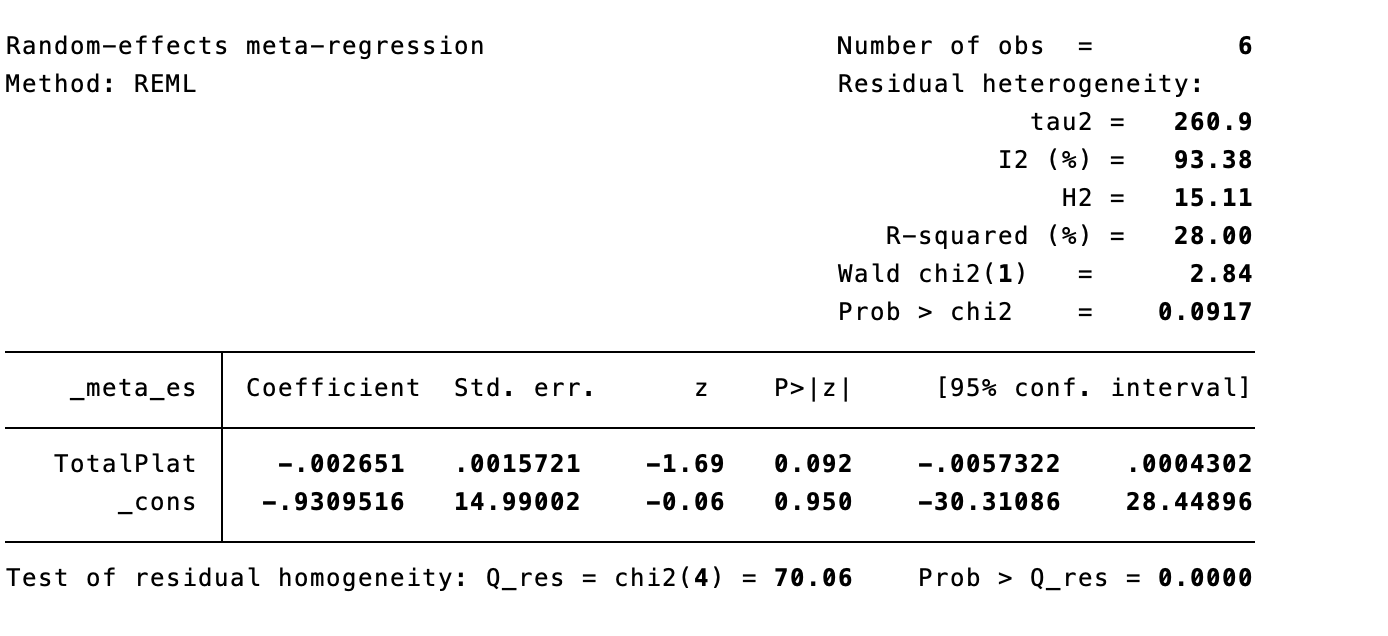

Supplement: Supplementary file 1 — Supplementary Material 1 [file 12178_2024_9922_MOESM1_ESM.docx]
